# The Influences of Sulphation, Salt Type, and Salt Concentration on the Structural Heterogeneity of Glycosaminoglycans

**DOI:** 10.3390/ijms222111529

**Published:** 2021-10-26

**Authors:** Suman Samantray, Olujide O. Olubiyi, Birgit Strodel

**Affiliations:** 1 Institute of Biological Information Processing: Structural Biochemistry (IBI-7), Forschungszentrum Jülich, 52428 Jülich, Germany; s.samantray@fz-juelich.de (S.S.); olubiyioo@oauife.edu.ng (O.O.O.); 2 AICES Graduate School, RWTH Aachen University, Schinkelstraße 2, 52062 Aachen, Germany; 3 Department of Pharmaceutical Chemistry, Faculty of Pharmacy, Obafemi Awolowo University, Ile-Ife 220282, Nigeria; 4 Institute of Drug Research and Development, Afe Babalola University, Ado-Ekiti 361212, Nigeria; 5 Institute of Theoretical and Computational Chemistry, Heinrich Heine University Düsseldorf, 40225 Düsseldorf, Germany

**Keywords:** glycosaminoglycans, sulphation, GAG–cation interactions, conformational flexibility, molecular dynamics simulations

## Abstract

The increasing recognition of the biochemical importance of glycosaminoglycans (GAGs) has in recent times made them the center of attention of recent research investigations. It became evident that subtle conformational factors play an important role in determining the relationship between the chemical composition of GAGs and their activity. Therefore, a thorough understanding of their structural flexibility is needed, which is addressed in this work by means of all-atom molecular dynamics (MD) simulations. Four major GAGs with different substitution patterns, namely hyaluronic acid as unsulphated GAG, heparan-6-sulphate, chondroitin-4-sulphate, and chondroitin-6-sulphate, were investigated to elucidate the influence of sulphation on the dynamical features of GAGs. Moreover, the effects of increasing NaCl and KCl concentrations were studied as well. Different structural parameters were determined from the MD simulations, in combination with a presentation of the free energy landscape of the GAG conformations, which allowed us to unravel the conformational fingerprints unique to each GAG. The largest effects on the GAG structures were found for sulphation at position 6, as well as binding of the metal ions in the absence of chloride ions to the carboxylate and sulphate groups, which both increase the GAG conformational flexibility.

## 1. Introduction

Glycosaminoglycans (GAGs), with their presence in the extracellular matrix [1], blood vessels, and tissues in the human body, play an important role in cellular signaling activity, anticoagulation, and angiogenesis and have been implicated in various other biological processes [2,3]. They are large unbranched polymers of differentially sulphated carbohydrate units often found covalently attached to proteins (except hyaluronic acid) in proteoglycans. Their ubiquitous presence in the extracellular matrix as much as on cell surfaces points to a primary role in cellular communication and adhesion and other biological processes dependent on these two, including cell recognition of growth factors, chemokines, adhesion molecules, and enzymes [4]. While describing the full interactome of GAGs is an ongoing venture, other molecular systems they interact with include serpins involved in anticoagulation; cytokines involved in immune regulation, inflammation, and blood development; matrix proteins for structural stability; protein receptors in the control of receptor dimerization; lipoproteins involved in lipid translocation; plaque proteins implicated in neurodegeneration; as well as complement structures in viral pathogens needed for entry into the host cell [5].

An inspection of the GAG interactome indicates their involvement in more than just physiological processes: they have been found to play a role in various pathological conditions. For instance, their interaction with amyloidogenic proteins implicates them as participants in the pathogenesis of amyloid-related neurodegenerative disorders, such as Alzheimer’s disease, type-2 diabetes, Parkinson’s disease, and prion diseases [6,7]. Their involvement in several biomolecular processes has carved out a niche for GAGs in clinical therapeutics. For example, chondroitin and hyaluronate are used in different pharmaceutical and nutraceutical formulations for the treatment of osteoarthritis [8,9,10,11]; heparin and its mimetics are used as anticoagulants for treating thrombosis; keratan sulphate is incorporated in ophthalmic products for treating certain eye defects and as biomarker for female genital tract carcinoma [12]. Beyond their use in different capacities as a replacement therapy, recent studies have suggested additional roles as possible therapeutic agents in inhibiting cell invasion by viruses causing severe acute respiratory syndrome (SARS) diseases [13,14].

It is crucial to point out that in all their areas of relevance—physiological, pathological, or therapeutics—the participation of GAGs results principally from their unique physicochemical and structural features, including high negative charge, high viscosity and lubricative attributes, unbranched polysaccharide structures, low compressibility, as well as the ability to attract and imbibe large amounts of water. Because GAGs are highly heterogeneous, they pose great challenges to research investigations. First, variable building units are involved in assembling the polymers, whose final lengths can vary widely. GAGs are also differentially sulphated, an event that is influenced by a host of factors including age, physiological conditions, and diseases. Their structural complexity is further extended by attachment to sugar and protein units.

GAGs behave as a hydrophilic polyelectrolyte, whereby they form swollen random coils in an aqueous environment [15,16,17]. However, such a structural preference of GAGs is modified under the influence of salts differing in type and concentration. For example, the ion concentrations in solution influence the size and shape of hyaluronic acid’s (HA) random coils, allowing site-specific HA–ion interactions via the decay of hairpin-like loops [18]. The ionic interactions between the cations and specific sites on the GAG surface are influenced by sulphation of the monosaccharide units of GAGs, such as glucosamine in heparan-6-sulphate (H6S) and galactosamine in chondroitin-4-sulphate (C4S) and chondroitin-6-sulphate (C6S) [19,20]. The sulphation pattern encoded in the GAG sequences acts as a molecular recognition tool to identify growth factors and allow activation of connected signaling pathways. Apart from the nonspecific electrostatic interactions in complex environments, GAGs also display ion-specific interactions by behaving as a biochemical barrier permeable only to monovalent and divalent ions [21], such as Na${}^{+}$, K${}^{+}$, Ca${}^{2+}$, and Mg${}^{2+}$ [22,23]. A detailed understanding of the sulphate moieties in controlling their intramolecular and intermolecular interactions, as well as their interactions with cations would be key for designing GAG-based therapeutic agents to induce their binding affinity to other biomacromolecules. Taken together, arriving at a common structural description for a biomolecular system so complex, or even determining their conformational preferences by traditional structure determination methods has been nothing short of daunting.

To characterize the GAGs’ behavior in biological processes, it is paramount to develop an understanding of which specific structural patterns they adopt under different physiological conditions and how they do so [24,25]. In our work, we provide crucial insight into the structure and dynamics of various GAGs using molecular dynamics (MD) simulations. MD simulations have been successfully used in the past to approach this problem. For instance, in various simulation studies, Guvench and coworkers elucidated in detail the effects of the sulphation pattern and the binding of Na${}^{+}$, Ca${}^{2+}$, or Mg${}^{2+}$ on chondroitin and other GAGs [26,27,28,29]. By combining two-dimensional infrared (IR) spectroscopy experiments with MD simulations, Bakker and coworkers showed that the Ca${}^{2+}$ binding to HA increases the flexibility of this GAG [30]. Other biophysical approaches for studying GAGs include X-ray crystallography, of GAGs alone [31,32] or in complex with proteins [33,34,35,36,37,38], as well as solution nuclear magnetic resonance (NMR) spectroscopy [39,40]. Compared to experimental techniques, MD simulations are an attractive and relatively inexpensive tool for exploring conformational transitions in GAGs at a microsecond time scale and with atomic resolution [41,42,43,44]. Simulations have been used extensively to study the conformational plasticity of different GAGs including hyaluronan [45], chondroitin sulphate [46], and heparan sulphate [47]. The structural diversity of GAGs coupled with GAG–protein binding interactions using multiscale modeling approaches was highlighted in recent reviews [48,49]. By modeling various physiological conditions in this study, we were able to present insight into how changes in these conditions affect the structures and dynamics of HA, H6S, C4S, and C6S. A heuristic method is provided to predict the free energy landscape characterizing the conformational ensembles of the GAGs.

## 2. Materials and Methods

### 2.1. Model Systems

**Figure 1:**
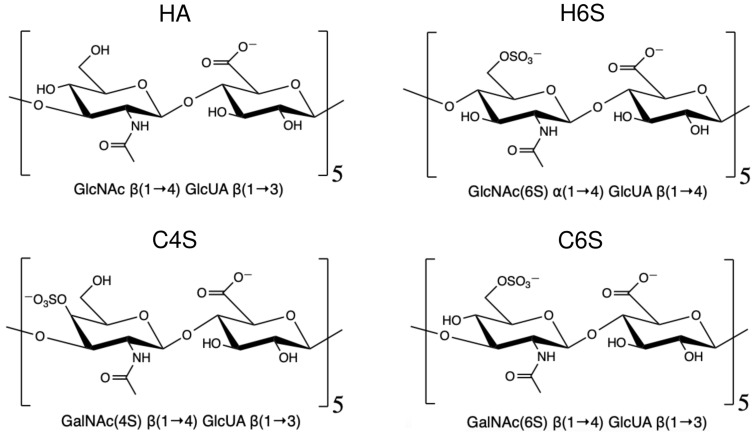
Chemical structure of the GAGs considered in this work. The disaccharide unit of each GAG is shown, and five repeating disaccharides were simulated in each case.

To understand the effects of different salts and salt concentrations on the structure and dynamics of different GAGs, we simulated the following five repeating disaccharide units: HA as a nonsulphated GAG and H6S, C4S, and C6S as sulphated GAGs (Figure 1). The repeating disaccharide units for HA involve D-glucosamine (GlcNAc) and D-glucuronic acid (GlcUA) linked via alternating β-(1→4) and β-(1→3) glycosidic bonds (O-linked): -GlcNAc-β(1→4)-GlcUA-β(1→3)-. In H6S, the disaccharide building blocks are identical to those in HA apart from sulphation at position 6 of GlcNAc and glycosidic linkages with different geometries: -GlcNAc(6S)-α(1→4)-GlcUA-β(1→4)-. C4S and C6S are composed of similar components and glycosidic linkage patterns as HA, except for GlcNAc being replaced by D-galactosamine (GalNAc). The difference between C4S and C6S exists in the sulphation points on the GalNAc unit, which is at carbon 4 in C4S and position 6 of GalNAc in C6S, leading to -GalNAc(4S)-β(1→4)-GlcUA-β(1→3)- and -β(1→4)-GlcUA-β(1→3)-GalNAc(6S)-, respectively. The disaccharide units and linkages (referred to as $Linkage_{1}$ and $Linkage_{2}$) are summarized in Table 1.

### 2.2. Simulation Protocols

We simulated the penta-disaccharide GAG units by placing their extended structures in the simulation box (Figure S1), making sure that the minimum distance between the corresponding GAG structure and any edge or face of the cubic box was at least 1.2 nm. For modeling the GAG molecules, we employed the most current version of the all-atom CHARMM36 force field developed for carbohydrates [50,51,52,53]. The CHARMM parameters are available in the “Glycan Reader & Modeler” module [54,55,56] of the CHARMM-GUI web server [57] as part of the “toppar_c36_jul20” force field package. This version of CHARMM36 also includes the latest pair-specific nonbonded Lennard–Jones (LJ) parameters for the Na${}^{+}$-COO${}^{-}$, Na${}^{+}$-OSO${}_{3}^{-}$, K${}^{+}$-COO${}^{-}$, and K${}^{+}$-OSO${}_{3}^{-}$ ion pairs, which were optimized by Yoo and Aksimentiev using osmotic pressure simulations [58]. These parameters are provided as so-called nonbonded FIX corrections (NBFIX) to the force field, which can be found in the file “nbfix.itp”. NBFIX is a possibility to adjust pair-specific LJ parameters without modifying solute–water interactions. In a previous simulation study of GAGs interacting with Na${}^{+}$ and K${}^{+}$, the same adjusted LJ parameters were employed [59]; they were listed in Table S1b in the Supporting Information of that previous work.

The resulting simulation box had an edge length of 6.8 nm, which was solvated with water using the TIP3P model [60] and resulted in about 32,000 atoms per system. We designed a set of four simulations for each GAG by employing different salts and salt concentrations. In the first system, Na${}^{+}$, but no Cl${}^{-}$, was added to neutralize the system. For simplicity, this system is denoted as “0 mM NaCl”, while in fact it contained about 25 mM Na${}^{+}$ in the HA setup and 50 mM Na${}^{+}$ in the case of H6S, C4S, and C6S. In the second system, a concentration of 150 mM NaCl was added. Two corresponding KCl systems were created and denoted 0 mM and 150 mM KCl, respectively. The number of ions required to neutralize each GAG system corresponding to their salt concentration and the total number of atoms per system are listed in Table 2. Each system was minimized using the steepest descent algorithm, followed by equilibration, first with a 20 ps run in the NVT ensemble with position restraints on the nonhydrogen atoms of the GAGs, afterwards with a 20 ps run in the NpT ensemble without position restraints. For the production runs, we simulated the GAG systems for 1 μs in the NpT ensemble at T=300 K and p=1 bar. Throughout the simulations, we constrained all bonds involving hydrogen atoms using the LINCS algorithm [61]. The electrostatic interactions were calculated using the particle mesh Ewald (PME) method [62], and their real-space components were truncated at 1.2 nm. The same cutoff was applied for the calculation of the van der Waals interactions. The temperature and pressure were controlled using a Nose–Hoover algorithm with a 1 ps time constant for coupling and a Parrinello–Rahman barostat [63] with a relaxation time of 5 ps, respectively. A time step of 2 fs was used for the integration of the equations of motion for all systems. All MD simulations were realized with GROMACS Version 2018.3 [64,65,66]. For the analysis of the simulation trajectories, we employed a combination of standard GROMACS tools, VMD [67], and in-house Python scripts [68] invoking the MDAnalysis [69] and MDTraj [70] libraries. The workflow to perform a dimensionality reduction, clustering based on internal GAG coordinates, and calculation of the free energy profile of the GAGs is available at https://github.com/suman-samantray/GAG-clustering-FES (accessed 22 July 2021).

### 2.3. Conformational Analysis

To quantify the flexibility of the GAGs, we calculated the root-mean-squared deviation (RMSD) by fitting the whole GAG structure to the initial configurations. To identify similar structures among the sampled conformations within a specified RMSD cutoff, we performed cluster analyses using the GROMACS clustering tool from Daura et al. [71].

This method is based on the RMSD between all conformations sampled during an MD simulation. An RMSD cutoff was chosen to determine cluster membership. To this end, the number of other snapshots that had an RMSD within RMSD_cutoff_ from each of the MD snapshots was counted, and the snapshot with the largest number of such neighbors was determined. This snapshot and its neighbors formed a cluster, which was eliminated from the pool of snapshots, and the procedure was repeated for the remaining MD samples until all snapshots were assigned to a cluster. Cutoff values of 0.3 nm, 0.4 nm, and 0.5 nm were tested. For both RMSD calculation and clustering, all GAG atoms were included in the computations. We also calculated contact formation between the GAGs’ monosaccharide units. In our analysis, two GAG residues were assumed to be in contact if the distance between any pair of atoms from residue *a* and residue *b* was 0.4 nm or less. The end-to-end distance ($R_{\mathrm{ee}}$) of the GAGs was determined by calculating the distance.

**Figure 2:**
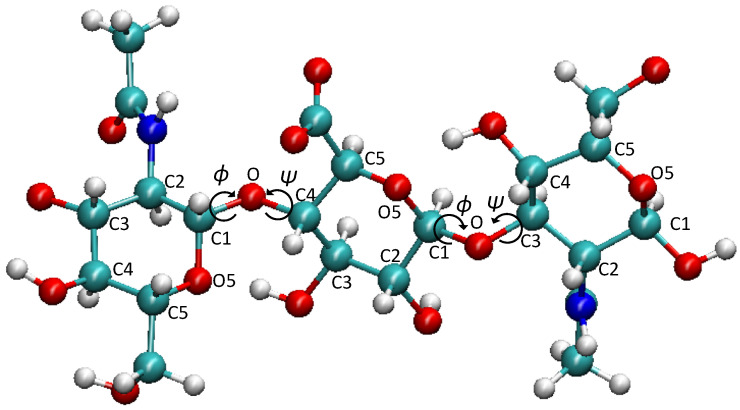
The definition of the dihedral angles ϕ and ψ is shown for HA.

Between the C1 atom of the starting residue (right residue in Figure 2) and the C4 atom of the terminal residue (left residue in Figure 2), to characterize the torsional motions of the GAG chains, we calculated the dihedral angles ϕ and ψ across the glycosidic linkage bonds. Their definitions are shown in Figure 2; the ϕ angle is defined by O5-C1-O4-C4 and is directly equivalent to the O5${}^{\prime }$-C1${}^{\prime }$-O4-C4 convention of the International Union of Pure and Applied Chemistry (IUPAC). In the case of the ψ, our force field lettering is C1-O4-C4-C3, which equals the C1${}^{\prime }$-O4-C4-C3 definition of the IUPAC. The only difference is the omission of the prime on atoms O5 and C1 [72].

The ϕ and ψ dihedral angles for each linkage type were combined into a single dihedral offset function, ($D_{\mathrm{offset}}$) [73], making it easier to capture deviations in both dihedral angles and across a GAG chain via a single descriptor:

(1) $$D_{\mathrm{offset}}=\frac{1}{N}\sum_{i=1}^{N}\left(1+\cos\left(\phi_{i}-\phi_{\mathrm{ref}}\right)\right)+\left(1+\cos\left(\psi_{i}-\psi_{\mathrm{ref}}\right)\right)$$

Here, $\phi_{\mathrm{ref}}$ and $\psi_{\mathrm{ref}}$ are the reference dihedral angles as present in the initial, fully extended GAG structures. The number *N* refers to the number of linkages found in each GAG, which is N=5 for $Linkage_{1}$ and N=4 for $Linkage_{2}$ for a GAG with five disaccharide units.

To gain a better understanding of the structural preferences of the GAG chains, we further calculated the number of hydrogen bonds formed between the GAG residues and water molecules ($N_{\mathrm{HB}}$). Here, a hydrogen bond was assumed to be present when the donor–acceptor distance was less than 0.35 nm and the donor-H-acceptor angle was less than $30^{\circ }$. We also characterized the interactions between the GAGs and the cations by calculating the radial distribution functions (RDFs) between Na${}^{+}$/K${}^{+}$ and the sulphate oxygen atoms (in GlcNAc of H6S and GalNAc of C4S and C6S), as well as the carboxylate oxygen atoms (in GlcUA of all GAGs). This was done for all five sulphate and five carboxylate groups present in the GAGs, after which the five corresponding profiles obtained were averaged.

### 2.4. Free Energy Landscape

In our effort to describe the collective modes of structural fluctuations observed in GAGs, we performed principal component analysis (PCA) on the previously calculated observables, specifically the $D_{\mathrm{offset}}$ for $Linkage_{1}$ and $Linkage_{2}$, $R_{\mathrm{ee}}$, and $N_{\mathrm{HB}}$. Based on the first two principal components (PCs), which capture the majority of the variance in our MD data, we built a 2D free energy landscape of the GAGs’ conformational space. The free energy was calculated by computing the probability distribution along the PCs, P(PC),

(2) $$\Delta G\left(PC\right)=-k_{B}T\ln\left(\frac{P(PC)}{P_{\max}}\right)$$

where $k_{B}$ is the Boltzmann constant and $P_{\max}$ denotes the maximum probability along the selected PC. The segregation of the conformational space into distinguishable cluster states projected onto the first two PCs was accomplished using k-means clustering. To determine the ideal number of clusters, we measured the sum of the squared distances to the nearest cluster center, i.e., the inertia, plotted the inertia for increasing cluster numbers, and applied the elbow method to the resulting curve.

## 3. Results and Discussions

### 3.1. Characterization of the GAGs’ Structural Data

Polymeric GAG chains in solution can assume a number of configurations that contribute to their entropy. For elucidation of the energetically stable structures of the GAGs, it is important to identify and segregate their conformational ensembles under diverse physiological conditions. In this section, we assess the role of salt ions in inducing conformational heterogeneity in the different GAGs and subsequently determine critical collective variables in order to describe the structural transitions.

#### 3.1.1. RMSD-Based Conformational Clustering

To determine the flexibility of the GAGs at different salt concentrations, we studied the time evolution of their RMSDs from the corresponding MD starting structure (Figure S2). The obtained RMSD variations were in the range of 0.4–0.6 nm, regardless of the salt type and concentration, which allowed us to conclude that the GAGs are quite flexible, especially when compared to folded proteins and considering their relatively short chain length. Some of the largest RMSD increases were seen for C4S, which could even reach values above 0.7 nm. To obtain an understanding of these structural fluctuations, we characterized the C4S structures with RMSD > 0.7 nm for each salt condition and report the various quantities, such as the end-to-end distance and dihedral angles ϕ and ψ in Table S1. The end-to-end distance was clearly smaller than the 4.8 nm of the fully extended structure, indicating that the RMSD increases result from C4S conformations deviating from linearity. This was confirmed by Figure S3, which shows representative C4S configurations with RMSD > 0.7 nm. They all possess curved shapes.

**Figure 3:**
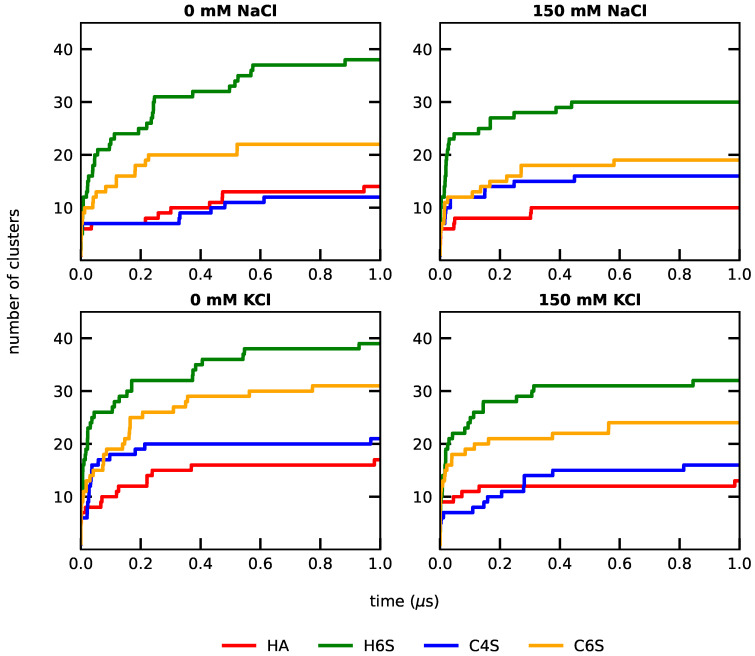
Evolution of the number of conformational clusters at RMSD_cutoff_ = 0.4 nm for the different GAGs (see the color key at the bottom) at the 0 mM (**left**) and 150 mM (**right**) salt concentrations (see the label above each panel).

A drawback of the RMSD analysis in Figure S2 is that it measured the deviation with respect to only one reference structure, which here is the GAG structure at the start of each MD simulation. Therefore, to further characterize the structural heterogeneity and also to assess sampling convergence, we performed RMSD-based geometric clustering using different cutoff values: RMSD_cutoff_ = 0.3, 0.4, and 0.5 nm (Figure S3, Figure 3, and Figure S4, respectively). Since the clustering was based on the RMSD between all structures sampled during a simulation, the number of clusters obtained was a direct measure of the flexibility of the molecule under study: the larger the number of clusters, the more flexible the molecule is. The results for the 0.4 nm cutoff showed that H6S had the highest number of clusters and was thus the most flexible GAG, followed by C6S, C4S, and HA (in this order). It is interesting to note that this trend was unaffected by salt type and concentration. HA, the least flexible of the lot, showed only limited dynamics and stabilized quickly without much sampling of unique configurations. This behavior contrasts with that of the sulphated GAGs. Sulphation at carbon 6, compared to carbon 4, generally increased the GAGs flexibility, but made H6S and C6S more susceptible to a higher salt concentration. At a 150 mM salt concentration, the conformational fluctuations of H6S and C6S were smaller in comparison to their behavior in the presence of small Na${}^{+}$ and K${}^{+}$ concentrations and missing Cl${}^{-}$ (denoted 0 mM salt). Another interesting observation was that C4S was overall less flexible than H6S and C6S, even though some of the highest RMSD peaks were recorded for C4S (Figure S2). This again showed that analyzing the RMSD with respect to only one reference structure was not sufficient for characterizing molecules as flexible as GAGs, which agrees with our findings made for intrinsically disordered peptides [74].

Clustering at RMSD_cutoff_ = 0.4 nm showed a convergence to a stable number of clusters for the different systems, which also indicated that the simulations reached convergence [74]. While running multiple independent simulations per system would add confidence to this conclusion, the graphs in Figure 3 obtained from single trajectories are different enough from each other to compare the results among each other—as just done—and to those obtained at other cutoff values. For a cutoff value of 0.3 nm, the number of clusters for the H6S system was rather high (Figure S4), as already minor structural changes were recorded here. This indicated that this cutoff value was too small to assess sampling convergence and structural diversity. On increasing the RMSD_cutoff_ to 0.5 nm (Figure S5), the number of clusters observed was greatly reduced, blanketing the diverse conformational classification of the GAGs.

#### 3.1.2. Intramolecular Interactions in GAGs

**Figure 4:**
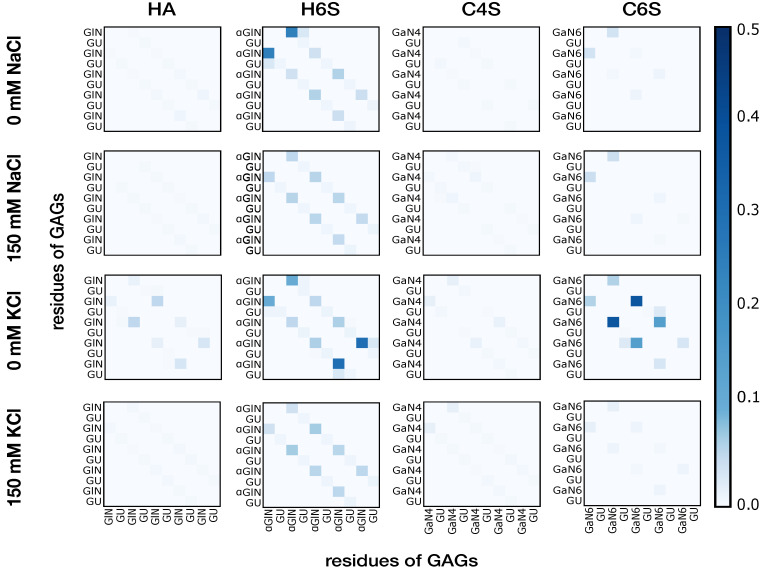
Intramolecular contacts among the residues of the GAGs at 0 mM NaCl, 150 mM NaCl, 0 mM KCl, and 150 mM KCl concentrations (labels left of each panel). The residues of the GAGs are abbreviated with the following codes: GU (GlcUA), GlN (GlcNAc in HA), αGlN (GlcNAc(6S) in H6S), GaN4 (GalNAc(4S)), and GaN6 (GalNAc(6S)). The color code on the right represents the probability of a contact among residues during the MD simulations. For the sake of clarity, the diagonal and first off-diagonal elements of the contact maps corresponding to self-contacts within the same disaccharide unit are not shown.

In order to elucidate the origin of the flexibility differences observed in the different investigated GAG molecules, we calculated the intramolecular contacts between individual residues (Figure 4). What can be immediately observed is that the interaction map was predominantly dominated by weak contacts. In the few cases with more pronounced contacts, mainly for H6S and C6S, these interactions were limited to disaccharide units in close proximity. For instance, for H6S at 0 mM NaCl, GlcNAc of a disaccharide unit was found to interact strongly with both GlcUA and GlcNAc of the adjacent disaccharide units. From the generally low contact probabilities, one can infer that the predominantly sampled structures were extended, but with a sufficient curvature that allowed contacts between neighboring residues. Interestingly, C4S, which was observed to have conformations with a high RMSD from the completely extended structure due to curvatures along the polymer chain (Figures S2 and S3), did not show pronounced intramolecular contacts. This can be explained by the fact that the curved C4S conformations occurred only rarely and therefore did not leave a prominent mark in the time-averaged contact probabilities. The observation that GAGs with sulphated moieties at carbon 6 had a tendency to form more contacts suggested that these intramolecular interactions increased their overall flexibility, as reported above (Figure 3). The contacts were stronger at 0 mM salt concentration than at 150 mM NaCl or KCl. For instance, some interactions were present in HA at 0 mM KCl, while they were missing at 150 mM KCl. In fact, for all simulated systems, most contacts were formed at 0 mM KCl.

#### 3.1.3. Shape of the GAGs

**Figure 5:**
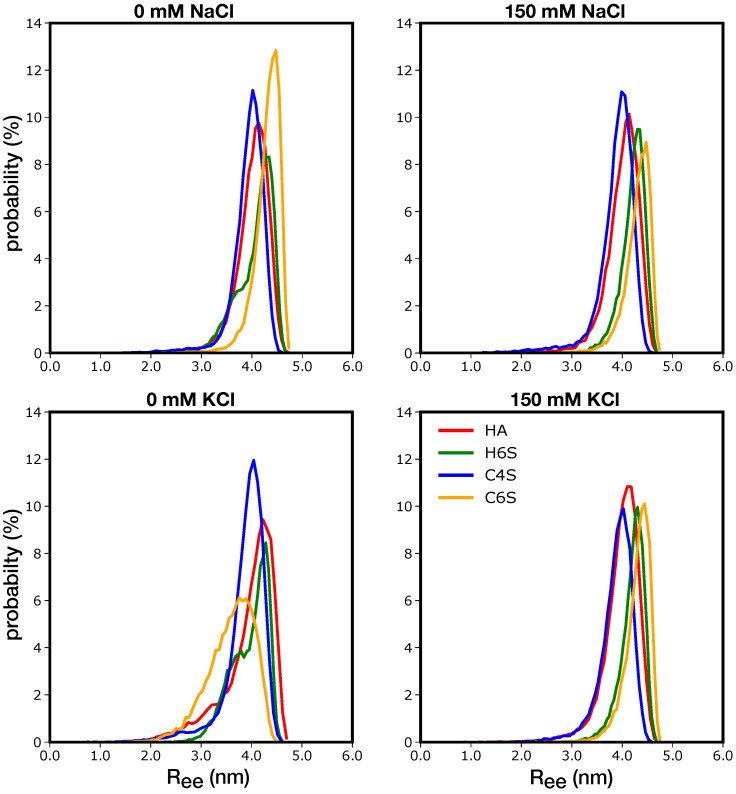
Distribution of the end-to-end distance ($R_{\mathrm{ee}}$) for the different GAGs (see the color key) at the 0 mM (**left**) and 150 mM (**right**) salt concentrations (see the label above each panel).

To further characterize the structural preferences of the GAGs, we calculated their end-to-end distance ($R_{\mathrm{ee}}$). The fully extended GAG molecules involving five disaccharide units had an $R_{\mathrm{ee}}$ of about 4.8 nm. The results in Figure 5 reveal that the GAGs simulated at different salt concentrations preferred to remain nearly extended with the main peaks of the $R_{\mathrm{ee}}$ distributions hovering around ∼4.0 nm. Nonetheless, these distributions also uncovered that at 0 mM salt, more compact GAG conformations exhibiting smaller $R_{\mathrm{ee}}$ values were possible. This was the case for H6S at both 0 mM NaCl and KCl, as well as HA, C4S, and C6S at 0 mM KCl. These were the same systems for which intramolecular contacts were identified (Figure 4), which indicates that the interactions among the saccharide units cause the GAGs to deviate from the extended geometry. The $R_{\mathrm{ee}}$ distributions for HA, H6S, and C4S at the indicated salt concentrations featured a second peak for smaller $R_{\mathrm{ee}}$ values, suggesting a switch between extended and more compact GAG conformations. For C4S, such a switch was observed in the RMSD jumps to values above 0.7 nm, which corresponded to curved C4S conformations (Figures S2 and S3). In the case of C6S at 0 mM KCl, on the other hand, the $R_{\mathrm{ee}}$ distribution became broader and was generally shifted to smaller values. This signaled a conformational preference for more compact structures stabilized by contacts between the second and third, as well as between the third and fourth disaccharide units. In the case of H6S, the strongest interdisaccharide contacts were formed at the GAG termini, i.e., between the first and second, as well as fourth and fifth disaccharide units.

The $R_{\mathrm{ee}}$ values observed here were in good agreement with the values previously reported for larger GAGs. For instance, Bakker and coworkers simulated HA with eight disaccharide units and obtained a time-averaged end-to-end distance of 0.7 nm, while the contour length was reported as 0.8 nm [30]. These lengths are roughly eight-/five-times those (resulting from eight versus five disaccharide units) that we recorded. Guvench and coworkers studied various sulphated and nonsulphated GAGs with either five or ten disaccharide units (called 10-mers and 20-mers, respectively) and found very similar $R_{\mathrm{ee}}$ distributions for the 10-mers [27,28] and distributions peaking at about 0.8 nm in the case of the 20-mers [28,29]. The $R_{\mathrm{ee}}$ values were thus roughly doubled when increasing the GAG length from five to ten disaccharide units; however, the $R_{\mathrm{ee}}$ distribution became clearly broader toward smaller end-to-end distances with increasing GAG chain length. This indicated that the flexibility of the GAGs increased with their size, an influence that was not studied here.

The shape of the GAGs was closely coupled to the dihedral angles on either side of the oxygen atoms linking adjacent monosaccharides: these are the dominant degrees of freedom in GAGs since they allow the monosaccharides to adopt different orientations relative to each other. In order to simplify the discussion of these torsional angles, we combined them into a collective offset function for each linkage type according to Equation (1). As reference angles we used those for the extended GAG structures. Therefore, $D_{\mathrm{offset}}=2$ corresponds to fully extended conformations, and values smaller than two indicate deviation from linearity and thus more compact structures. The results in Figure S6 show that the GAGs experienced limited flexibility across $Linkage_{1}$. Independent of the salt concentration, salt type, and GAG, the $D_{\mathrm{offset}}$ values were between 1.6 and 2.0. The smallest values were found for C4S, which agrees with the $R_{\mathrm{ee}}$ distributions, which also peaked at slightly smaller values compared to the other GAGs at the corresponding conditions. However, since we know from the contact maps that no noteworthy intramolecular contacts formed in C4S, we expected this particular GAG to be maximally extended and only slightly curved. We thus concluded that for $Linkage_{1}$ only, $D_{\mathrm{offset}}<1.6$ would indicate significant deviation from extended structures. For $Linkage_{2}$, such low values were reached for H6S (under all conditions, but 0 mM KCl) and C6S (at 0 mM KCl). These were the same systems that feature notable intramolecular contacts leading to more compact conformations. Special attention is needed for HA and H6S at 0 mM KCl where the $D_{\mathrm{offset}}$ values were limited to the range 1.6 and 2.0 for $Linkage_{2}$, despite the presence of intramolecular interactions. In the case of HA, as well as for C6S at 0 mM KCl and H6S under all conditions, but 0 mM KCl, a splitting of the $D_{\mathrm{offset}}$ distribution into two peaks could be observed. We reckoned this to imply that for $Linkage_{2}$, this split indicated the formation of more compact GAG conformations. In the case of H6S at 0 mM KCl, however, the $D_{\mathrm{offset}}$ function failed to capture the GAG structures deviating from linearity. Here, a more detailed inspection of the ϕ and ψ dihedral angles would be required if one wants to learn more about the individual glycosidic linkage configurations.

### 3.2. Characterization of the GAGs Interactions with Water and Ions

The structural preferences of the GAGs originate not only from their intrinsic properties, such as their chemical composition and sulphation, but also from their interactions with the environment. Here, the GAGs’ surrounding was provided by the aqueous solvent and ions. To explore their role on the GAGs structures, we analyzed the interactions between them here.

#### 3.2.1. GAG–Water Interactions

**Figure 6:**
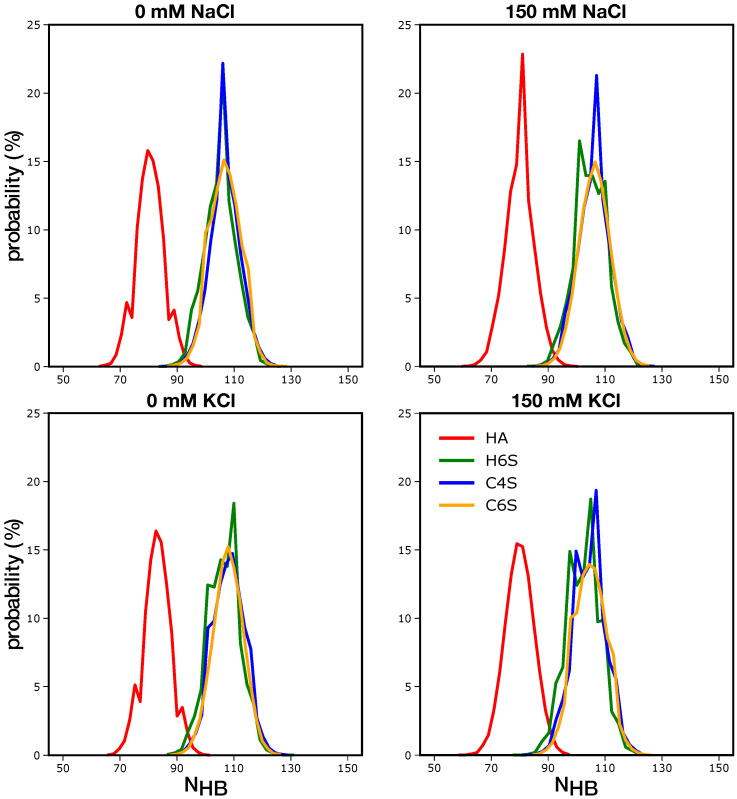
Distribution of the number of H-bonds formed between water molecules and the different GAGs (see the color key) at the 0 mM (**left**) and 150 mM (**right**) salt concentrations (see the label above each panel).

To characterize the GAG–water interactions, we calculated the number of hydrogen bonds (H-bonds) formed between GAGs and solvent molecules. In Figure 6, it can be observed that irrespective of the salt concentrations, HA formed the least number of such H-bonds. This can be easily explained with the presence of the sulphate (OSO${}_{3}^{-}$) group in the other three GAGs. A comparison between the H-bond numbers revealed that H6S, C4S, and C6S tended to build 20–30 H-bonds more with the surrounding water molecules than did HA. Since there are five OSO${}_{3}^{-}$ in each of H6S, C4S, and C6S, one can estimate that each sulphate group gives rise to about 4–5 H-bonds. This is probably slightly overestimated since H-bonds calculated were identified on geometric grounds only. Yet, the picture presented serves the purpose to demonstrate the significant effect that OSO${}_{3}^{-}$ has on GAGs’ binding of water molecules. The H-bond distributions of H6S, C4S, and C6S were very similar, and the GAG–water interactions did not help to explain the structural characteristics of the different GAGs.

#### 3.2.2. GAG–Ion Interactions

**Figure 7:**
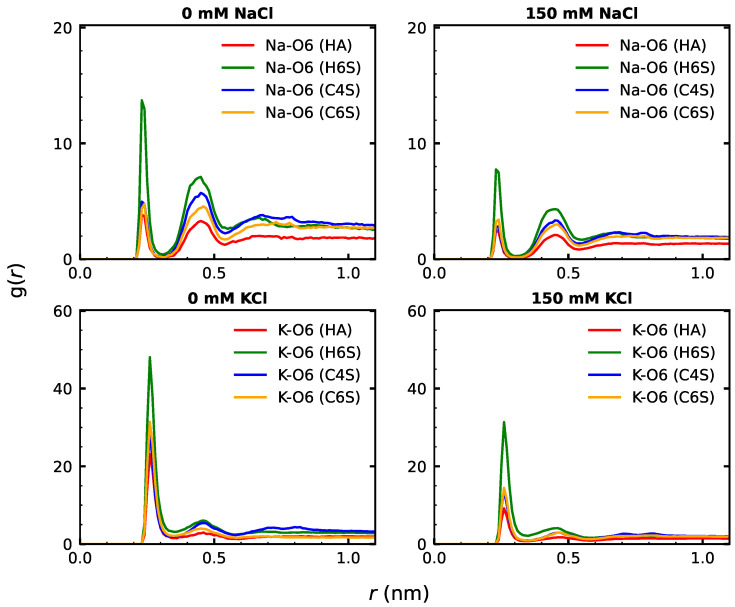
RDF curves representing the interactions between COO${}^{-}$ and the cations Na${}^{+}$ (**top**) and K${}^{+}$ (**bottom**) at the 0 mM (**left**) and 150 mM (**right**) salt concentration (see the label above each panel). The RDF curves were averaged over both oxygen atoms bound to carbon atom 6 (see Figure S6 for the atom naming) of GlcUA and over the five GlcUA units per GAG, in addition to averaging over the simulation time.

We next turn our attention to the interactions between the GAGs and the cations, K${}^{+}$ and Na${}^{+}$. Given the negative charge of the GAGs, there are no attractive interactions with the Cl${}^{-}$ ions that needed to be considered. As shown by others, cations prefer to localize near the OSO${}_{3}^{-}$ and COO${}^{-}$ groups of the GAGs (see Figure S7 for the atom naming of these groups), leading to the formation of contact ion pairs and solvent-separated ion pairs [59]. These pairs can be identified from the radial distribution functions (g(r)), which are shown in Figure 7 for COO${}^{-}$ and in Figure S8 for OSO${}_{3}^{-}$. The cation–anion RDFs typically displayed two peaks: a larger one corresponding to direct cation–anion contacts at distances between 0.2 and 0.3 nm and a smaller one at a distance of 0.4–0.5 nm for the water-separated ion pair.

Comparison of the large peaks for the contact ion pairs uncovered three key differences among the systems. First, the direct cation–anion interaction involving K${}^{+}$ was generally stronger than for Na${}^{+}$. This difference was especially pronounced for the interaction with COO${}^{-}$. This finding was somewhat surprising, as the more strongly hydrated cations (here, Na${}^{+}$) usually bind more tightly to carboxylate groups than do the less hydrated cations (here K${}^{+}$) [75], which can be rationalized by the fact that Na${}^{+}$ matches better the hydration enthalpy of COO${}^{-}$ [76]. However, inspection of the second peak showed that the water-separated Na${}^{+}$–COO${}^{-}$ pair was stronger than the corresponding K${}^{+}$–COO${}^{-}$ pair. In fact, if one calculates the number of ions that were located in the vicinity of the GAGs (i.e., within 0.5 nm) during the simulations, the results in Figure S9 show that in the case of C6S at 0 mM NaCl, this number fluctuated around 3 Na${}^{+}$ ions, whereas it was only ∼2 for K${}^{+}$ at 0 mM KCl. For the other three systems, these numbers were similar for Na${}^{+}$ and K${}^{+}$, which indicated a similar preference of these GAGs for both ions. The analysis in Figure S9 further confirmed that the ion parameters that we used (as implemented in the toppar_c36_jul20 force field package) did not lead to an overestimation of the cation–anion interactions that was observed for Ca${}^{2+}$ when using the older ion parameters available in toppar_c36_jul17 [29], as in our study, less than 30% of the cations were close to the GAGs in the 0 mM salt systems, which even dropped to about 10% in the 150 mM salt systems. We further checked that there was a constant exchange among the cations that were in direct contact with the GAGs. The comparison of our RDF results for C4S at 150 mM NaCl in Figure 7 with those obtained by Guvench and Whitmore for a C4S 20-mer simulated with 140 mM NaCl (see the fourth row of Figure 8 of [29]) revealed very similar heights and widths for the first two peaks in both studies. This confirmed that Na${}^{+}$ has a high tendency to build solvent-separated ion pairs with COO${}^{-}$. The preference of K${}^{+}$ to form direct contacts with negatively charged oxygen atoms, on the other hand, was also detected in a simulation study of RNA involving K${}^{+}$ [77].

In order to understand the prevalence of solvent-separated ion pairs involving Na${}^{+}$, we performed a literature search for comparable studies and performed an in-depth visual inspection of the different trajectories. Sterling and coworkers explained this finding by the fact that a cation is often shared between two neighboring anions, which due to ion size and GAG rigidity limitations, can in some cases be better realized via solvent-separated ion pairs [59]. Here, we found that the smaller size of Na${}^{+}$ (ionic radius of 0.116 nm) compared to K${}^{+}$ (ionic radius of 0.152 nm) plays a role in that the former is more often found in solvent-separated ion pairs, as the comparison of typical binding modes found for H6S in Figure S10 demonstrated. Moreover, we observed that K${}^{+}$ prefers direct binding via COO${}^{-}$–K${}^{+}$–COO${}^{-}$ bridging, whereas Na${}^{+}$ was more often found in solvent-separated COO${}^{-}$–K${}^{+}$–OSO${}_{3}^{-}$ bridging. Both cations were equally involved in direct binding via COO${}^{-}$–cation–OH bridging, where the hydroxy group is part of the GlcNAc saccharide.

The second difference between the different systems is that the carboxylate group interacted more strongly with either cation than the sulphate group did; this agrees well with previous findings [26,29,59]. Finally, in the absence of Cl${}^{-}$ ions, i.e., in the 0 mM KCl and NaCl systems, the interactions between either cation and either of COO${}^{-}$ and OSO${}_{3}^{-}$ appeared stronger than in the presence of Cl${}^{-}$ ions (i.e., in the 150 mM KCl and NaCl systems). This conclusion can, however, not be drawn from the heights of the RDF peaks, which were smaller in the 150 mM systems. However, it should be considered that the RDF was normalized with respect to the density of the cation in question, which was obviously higher in the 150 mM systems. Therefore, a comparison of the RDF peak heights at different salt concentrations might be misleading. When assessing the number of cations in the GAGs’ vicinity, Figure S9 shows that this number was higher in the 150 mM systems. However, it did not increase by a factor of four (or seven) when the number of cations was being increased from ten to forty (or from five to thirty-five in the case of HA), but only by about two to three. Therefore, the conclusion was that in the absence of Cl${}^{-}$, the relative attraction between the GAGs and the cations was stronger.

The analysis of the interactions of the GAGs with the cations permitted identifying their effects on the GAGs’ structural preferences, especially when focusing on COO${}^{-}$ (Figure 7). The H6S group displayed the highest interaction preference for both cations and salt concentrations. It should be recollected that H6S is the same GAG with the highest level of intramolecular contacts. We can thus conclude that the GAG–ion interactions enabled more conformational flexibility in the GAGs. This resulted from a screening of their negative charges from each other, reducing the tendency of the GAGs to adopt extended structures where maximum distances between the COO${}^{-}$ and OSO${}_{3}^{-}$ groups can be reached. Indeed, a similar pattern of reduction in hyaluronan chain rigidity upon Ca${}^{2+}$ and Na${}^{+}$ binding was recently reported [30]. The binding of Ca${}^{2+}$ in particular was found to weaken intramolecular hydrogen bonds, which eventually caused increased chain flexibility. This observation was drawn from the joined application of linear and nonlinear IR spectroscopy, as well as MD simulations [30]. Other simulation studies also found that Ca${}^{2+}$ binding alters the GAG dynamics, giving rise to more compact conformations [26,29]. As H6S, also C6S showed both a stronger interaction with cations at 0 mM KCl compared to the other three salt conditions and the largest number of intramolecular contacts at 0 mM KCl. The same trend was seen with HA even though HA has no sulphate group, and the effect was therefore reduced. Only C4S did not follow this trend. It is generally less flexible—apart from the rarely sampled curved structures shown in Figure S3—yet displayed similar interaction patterns with Na${}^{+}$ and K${}^{+}$ as C6S did. Thus, the conformational freedom of a GAG is an intricate interplay between the position of the sulphate group and external conditions.

### 3.3. Free Energy Profile Based on Collective Fluctuations

**Figure 8:**
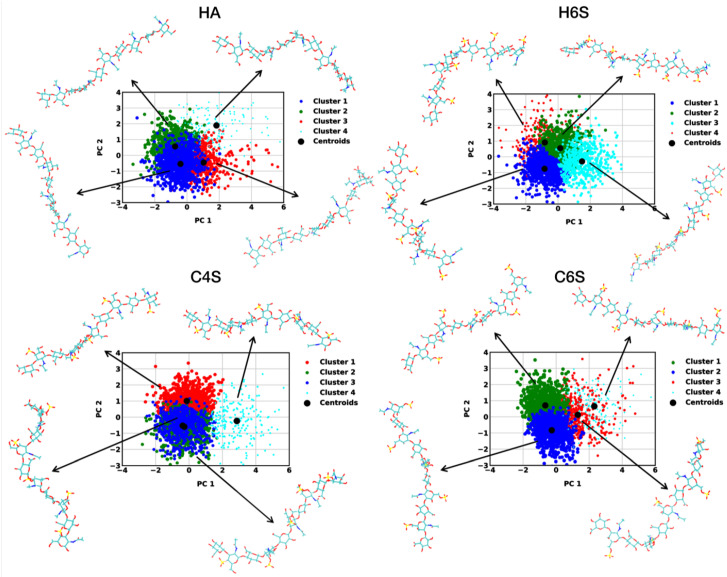
Projection of the MD trajectories obtained at 150 mM NaCl onto the first two principal components for the different GAGs (see the label above each panel). The conformations are segregated into four clusters, which are represented by different colors. Structures corresponding to the centroid structures are shown.

As we studied the behavior of GAGs with a wide variety of calculated structural observables, we now present an approach to disentangle the conformational states by a free energy method. We started by concatenating four of the previously introduced features, namely $D_{\mathrm{offset}}$ for $Linkage_{1}$ and $Linkage_{2}$, $R_{\mathrm{ee}}$, and $N_{\mathrm{HB}}$, into one state vector, which represents the structural evolution of the GAGs. As an example, the projection of all MD snapshots sampled for the GAGs at 150 mM NaCl is shown in Figure S11. A PCA was applied to this state vector, and the first two principal components (PCs) were chosen for calculating the free energy according to Equation (2). The resulting free energy surfaces for the 150 mM NaCl systems (Figure S12) revealed one main energy basin for each GAG. The width of these basins was somewhat larger for C6S and H6S, which is in agreement with their increased flexibility identified by the clustering analysis (Figure 3). The corresponding projection of all MD snapshots onto the first two PCs can be seen in Figure 8. In order to identify representative structures, we performed a k-means clustering of the structures. To decide on the numbers of clusters (*k*) to consider, we plotted the inertias for increasing *k* (Figure S13) and applied the elbow method to it. From this, we disclosed that four clusters were sufficient in segregating the conformational space, since for k>4, the inertias reduced only mildly. The centroids of the four clusters were projected along the first two PCs, and representative GAG structures per cluster centroid were retrieved (Figure 8). In Table 3, the structural characteristics of these centroid structures are summarized. In the Supplementary Materials, the corresponding plots and tables are provided for the three other salt conditions: 0 mM NaCl, 0 mM KCl, and 150 mM KCl (Figures S14–S16 and Tables S2–S4).

Inspection of the cluster structures in Figure 8 supports the conclusions drawn from the contact map analysis (Figure 4). First, the GAGs had a high preference to adopt extended conformations, as the structures representing the most populated clusters for each of the GAGs fell into this category. For HA, H6S, and C6S, the $R_{\mathrm{ee}}$ of the first two centroid structures was above 4.2 nm and for C4S above 3.9 nm. Structures with smaller end-to-end distances were sampled with lower probabilities and were included in Cluster 4 of all four GAGs. For C4S, we already identified conformations that belong to Cluster 4 from the RMSD analysis, as they were characterized by a large deviation from the fully extended structure used as a reference in that analysis (Figure S3 and Table S1). These more compact structures featured curves and kinks along the GAG chain. The kinks can lead to perpendicular orientations, especially when they occur after two or three disaccharide units, while at the termini, the kinks led to hairpin-like turns. Such deviations from linearity can be stabilized by cation bridging where Na${}^{+}$ or K${}^{+}$ binds to the anionic or hydroxy groups of saccharide units that are not directly neighboring. Examples for such bridging modes can be seen in Figure S10. Another motion that can lead to kinks in the GAGs is a change in the ring pucker, as was discussed in detail by Guvench and coworkers [27]. Changes in the ring pucker occur predominantly in the GlcUA rings. The preferred ring conformation is the chair conformation, which places all exocyclic functional groups in an equatorial orientation. However, skew boat and boat ring puckers can be adopted as well, yet with lower probability, where one of the glycosidic linkage oxygen atoms is in the equatorial position and the other one is in the axial position. This leads to an approximately perpendicular orientation of the the O-C1 and C4-O bond vectors and, thus, a kink in the polymer chain [27]. An interesting observation noted in Table 3 is that the average $N_{\mathrm{HB}}$ value was greater for the highest populated cluster states for all GAGs. This is understandable considering that these are the most expanded GAG structures that have a larger solvent-accessible surface area than more compact structures. This analysis indicates that, while the $N_{\mathrm{HB}}$ distributions shown in Figure 6 did not help much to gain a deeper understanding of the GAGs conformational preferences, combining this analysis with structural clustering did help to correlate the different structural aspects with each other.

Inspection of the free energy profiles for the systems at 0mM KCl (Figure S15) revealed that under this condition, even more compact structures were possible, which were also sampled with a higher probability. For C6S, for example, a spherical conformation for C6S is shown in Figure S15. The corresponding numbers in Table S3 reveal that for HA, H6S, and C6S, the structures with the smallest end-to-end distances were now included in Cluster 3 (and not in Cluster 4 as at 150 mM NaCl), which were populated >20% of the time. This is in line with the contact analysis in Figure 4, which revealed that among all salt conditions, the most contacts among saccharide units and thus compact conformations formed at 0 mM KCl. At both 0 mM NaCl and 150 mM KCl, compact structures were sampled with lower probability compared to at 0 mM KCl, as Figures S14 and S16, as well as Tables S2 and S4 reveal. This again indicated the smaller effect that Na${}^{+}$ has on the GAGs, which agrees with the findings by Guvench and Whitmore, who revealed that Ca${}^{2+}$ has a stronger influence than Na${}^{+}$ to cause compact chondroitin conformations [29]

## 4. Conclusions

We investigated the structural dynamics of four GAGs—hyaluronic acid (HA), heparan-6-sulphate (H6S), chondroitin-4-sulphate (C4S) and chondroitin-6-sulphate (C6S)—using microsecond-long MD simulations and modeling different salt conditions. This allowed us to assess the influences of GAG composition and glycosidic linkage, sulphation, and external situations on their conformational ensembles. To quantify the GAGs’ dynamics, different quantities, such as the RMSD, the end-to-end distance, and the dihedral angles across the glycosidic linkages were determined. Moreover, to rationalize the GAGs’ structural preferences, we analyzed their interactions with water and the metal ions present in the systems. Finally, a method was presented that allowed us to efficiently determine the preferred GAGs structures and locate them in the conformational free energy landscape.

A general conclusion is that the GAGs have an intrinsic preference for extended structures. Deviation from linearity is enabled by sulphation, especially at position 6, and interaction with cations. The latter screen the negative charges that are next to each other in the GAGs, which allows the COO${}^{-}$ and OSO${}_{3}^{-}$ groups to become closer to each other, causing kinks and bends in the GAGs. If other anions, such as Cl${}^{-}$, were present in the system, this screening was reduced. As a result, H6S and C6S in the presence of only Na${}^{+}$ or K${}^{+}$ (but no Cl${}^{-}$) are the most flexible, while HA and C4S in the presence of 150 mM salt are the stiffest. The higher flexibility represents itself in more contacts between the disaccharide units of C6S and especially H6S. The differences between C6S and H6S highlight that also the GAG sequence and glycosidic linkage matter with respect to their conformational flexibility. The metal ions usually adopt a bidentate configuration when binding to the GAGs, where they interact with two anions at the same time. In order to realize such bidentate binding, the small Na${}^{+}$ must often interact with COO${}^{-}$ or OSO${}_{3}^{-}$ with a water molecule between them. This explains the smaller effect that Na${}^{+}$ has on the GAGs as compared to K${}^{+}$. However, it should be noted that, while we took care to use the latest ion parameters available within CHARMM36, these models neglect electronic polarization and might therefore suffer from an unbalanced representation of the GAG–ion interactions. Different approaches exist to overcome this deficiency of the classical ion models [78,79], which should be applied in the future in the context of GAG simulations. Finally, we revealed that the OSO${}_{3}^{-}$ groups have a large effect on attracting water to the GAGs, as the comparison of the GAGs–water interactions for HA and the three sulphated GAGs showed.

In summary, our simulation study highlighted certain key features of the structural dynamics of GAGs as influenced by cation binding and sulphation. Detailed knowledge thereof is needed if one aims to correlate GAG sequence with activity.

## Figures and Tables

**Table 1:** The glycosidic linkage models of the GAG systems.

System	${\mathrm{Linkage}}_{1}$	${\mathrm{Linkage}}_{2}$
HA	(-GlcNAc-β(1→4)-GlcUA-)	(-GlcUA-β(1→3)-GlcNAc-)
H6S	(-GlcNAc(6S)-α(1→4)-GlcUA-)	(-GlcUA-β(1→4)-GlcNAc(6S)-)
C4S	(-GalNAc(4S)-β(1→4)-GlcUA-)	(-GlcUA-β(1→3)-GalNAc(4S)-)
C6S	(-GalNAc(6S)-β(1→4)-GlcUA-)	(-GlcUA-β(1→3)-GalNAc(6S)-)

**Table 2:** Overview of the simulated systems.

System	0 mM NaCl	150 mM NaCl	0 mM KCl	150 mM KCl
HA	5 Na${}^{+}$, 0 Cl${}^{-}$	35 Na${}^{+}$, 30 Cl${}^{-}$	5 K${}^{+}$, 0 Cl${}^{-}$	35 K${}^{+}$, 30 Cl${}^{-}$
H6S	10 Na${}^{+}$, 0 Cl${}^{-}$	40 Na${}^{+}$, 30 Cl${}^{-}$	10 K${}^{+}$, 0 Cl${}^{-}$	40 K${}^{+}$, 30 Cl${}^{-}$
C4S	10 Na${}^{+}$, 0 Cl${}^{-}$	40 Na${}^{+}$, 30 Cl${}^{-}$	10 K${}^{+}$, 0 Cl${}^{-}$	40 K${}^{+}$, 30 Cl${}^{-}$
C6S	10 Na${}^{+}$, 0 Cl${}^{-}$	40 Na${}^{+}$, 30 Cl${}^{-}$	10 K${}^{+}$, 0 Cl${}^{-}$	40 K${}^{+}$, 30 Cl${}^{-}$

**Table 3:** The values of $R_{\mathrm{ee}}$, $N_{\mathrm{HB}}$, phi, and ψ pairs for $Linkage_{1}$ and $Linkage_{2}$ of the centroid structures of the four clusters (populations are provided below) obtained for the GAGs at 150 mM NaCl. The corresponding values of the initial structures used in the MD simulations are provided as well (mean ± standard error).

System	Structure	%	$R_{\mathrm{ee}}$(nm)	$N_{\mathrm{HB}}$	${\mathrm{Linkage}}_{1}$(°)	${\mathrm{Linkage}}_{2}$(°)
HA	starting		4.5	83.0	(−132.1, −146.1)	(−93.4, 76.1)
	cluster 1	34.8	4.2 ± 0.0	84.9 ± 0.1	(−74.5 ± 0.2, −128.2 ± 0.2)	(−77.2 ± 0.2, 124.3 ± 0.3)
	cluster 2	34.6	4.2 ± 0.0	76.3 ± 0.1	(−73.8 ± 0.2, −128.7 ± 0.2)	(−78.6 ± 0.2, 124.6 ± 0.3)
	cluster 3	25.0	3.8 ± 0.0	81.5 ± 0.2	(−67.7 ± 0.2, −119.7 ± 0.4)	(−78.9 ± 0.3, 129.3 ± 0.2)
	cluster 4	05.6	3.4 ± 0.1	81.8 ± 0.4	(−71.5 ± 0.5, −123.4 ± 0.8)	(−78.4 ± 0.6, 87.9 ± 1.3)
H6S	starting		4.5	102.00	(−111.6, 87.2)	(45.6, 64.3)
	cluster 1	32.3	4.3 ± 0.0	109.7 ± 0.2	(−70.9 ± 0.2, 123.9 ± 0.2)	(79.4 ± 0.4, 46.9 ± 1.0)
	cluster 2	31.4	4.3 ± 0.0	104.1 ± 0.2	(−76.8 ± 0.2, 116.3 ± 0.2)	(80.8 ± 0.3, 40.4 ± 1.0)
	cluster 3	22.2	4.4 ± 0.0	102.7 ± 0.2	(−73.4 ± 0.2, 119.1 ± 0.3)	(86.0 ± 0.3, −22.4 ± 0.9)
	cluster 4	14.1	3.8 ± 0.0	106.3 ± 0.3	(−73.4 ± 0.3, 120.7 ± 0.4)	(79.6 ± 0.5, 23.1 ± 1.8)
C4S	starting		4.4	108.0	(−139.4, −146.9)	(−102.8, 91.1)
	cluster 1	34.0	3.9 ± 0.0	111.1 ± 0.1	(−67.3 ± 0.2, −121.2 ± 0.2)	(−76.4 ± 0.2, 128.4 ± 0.3)
	cluster 2	30.7	4.0 ± 0.0	101.6 ± 0.1	(−67.6 ± 0.2, −121.6 ± 0.2)	(−78.5 ± 0.2, 125.5 ± 0.3)
	cluster 3	26.6	4.0 ± 0.0	109.0 ± 0.2	(−67.5 ± 0.2, −121.9 ± 0.2)	(−80.2 ± 0.2, 112.7 ± 0.5)
	cluster 4	08.7	3.2 ± 0.0	105.9 ± 0.4	(−52.3 ± 0.7, −119.0 ± 0.3)	(−78.7 ± 0.4, 114.8 ± 1.5)
C6S	starting		4.4	114.0	(−120.8, −156.3)	(−106.6, 87.4)
	cluster 1	38.8	4.4 ± 0.0	111.5 ± 0.1	(−74.3 ± 0.2, −140.6 ± 0.3)	(−72.0 ± 0.2, 118.6 ± 0.4)
	cluster 2	38.0	4.4 ± 0.0	103.3 ± 0.1	(−74.3 ± 0.2, −140.1 ± 0.2)	(−75.4 ± 0.2, 114.0 ± 0.4)
	cluster 3	17.6	3.9 ± 0.0	106.8 ± 0.2	(−69.8 ± 0.3, −133.4 ± 0.4)	(−75.6 ± 0.3, 111.4 ± 0.9)
	cluster 4	05.6	4.3 ± 0.0	106.3 ± 0.5	(−73.7 ± 0.8, −78.9 ± 1.3)	(−75.7 ± 0.5, 117.0 ± 0.9)

## Data Availability

The simulation trajectories are available from the authors upon request. All other data is presented in the main text or as Supplementary Materials.

## Supplementary Materials

The following are available at https://www.mdpi.com/article/10.3390/ijms222111529/s1.
