# Supplementary material for: The Influences of Sulphation, Salt Type, and Salt Concentration on the Structural Heterogeneity of Glycosaminoglycans
Source: Int J Mol Sci. 2021 Oct 26;22(21):11529. doi: 10.3390/ijms222111529 (PMC8583755; doi:10.3390/ijms222111529)
Supplement: Supplementary file 1 [file ijms-22-11529-s001.zip › ijms-1332156-supplementary.pdf]

International Journal of
Molecular Sciences

MDPI

Article

The Influences of Sulphation, Salt Type, and Salt Concentration on the Structural Heterogeneity of Glycosaminoglycans

Suman Samantray [1,2], Olujide O. Olubiyi [1,3,4] and Birgit Strodel [1,5,*]

[1] Institute of Biological Information Processing: Structural Biochemistry (IBI-7), Forschungszentrum Jülich, 52428 Jülich, Germany; s.samantray@fz-juelich.de (S.S.); olubiyioo@oauife.edu.ng (O.O.O.)
[2] AICES Graduate School, RWTH Aachen University, Schinkelstraße 2, 52062 Aachen, Germany
[3] Department of Pharmaceutical Chemistry, Faculty of Pharmacy, Obafemi Awolowo University, Ile-Ife 220282, Nigeria
[4] Institute of Drug Research and Development, Afe Babalola University, Ado-Ekiti 361212, Nigeria
[5] Institute of Theoretical and Computational Chemistry, Heinrich Heine University Düsseldorf, 40225 Düsseldorf, Germany
* Correspondence: b.strodel@fz-juelich.de

check for updates

Citation: Samantray, S.; Olubiyi, O.O.; Strodel, B. The Influences of Sulphation, Salt Type, and Salt Concentration on the Structural Heterogeneity of Glycosaminoglycans. *Int. J. Mol. Sci.* **2021**, *22*, 11529. https://doi.org/10.3390/ijms222111529

Academic Editors: Chiara Schiraldi, Donatella Cimini and Annalisa La Gatta

Received: 23 July 2021
Accepted: 14 October 2021
Published: 26 October 2021

Publisher's Note: MDPI stays neutral with regard to jurisdictional claims in published maps and institutional affiliations.

Copyright: © 2021 by the authors. Licensee MDPI, Basel, Switzerland. This article is an open access article distributed under the terms and conditions of the Creative Commons Attribution (CC BY) license (https://creativecommons.org/licenses/by/4.0/).

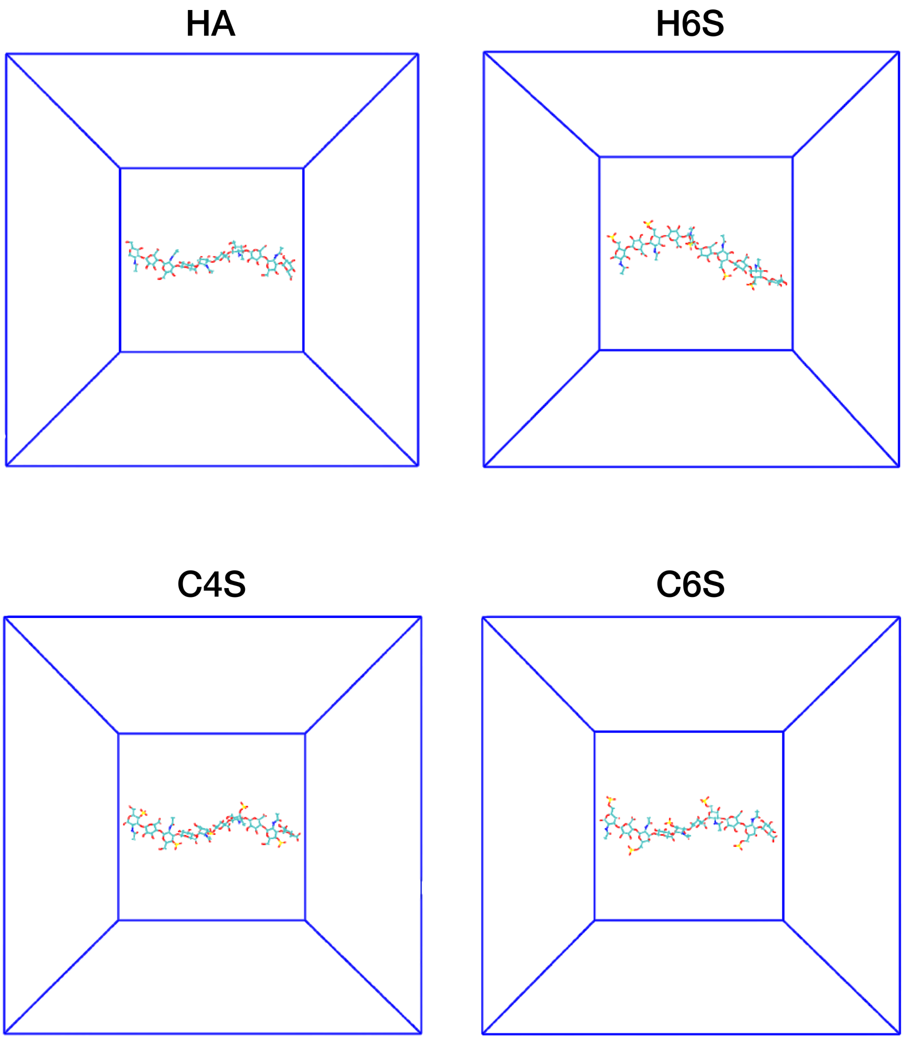

Figure S1. Initial GAG configurations used in the MD simulations.

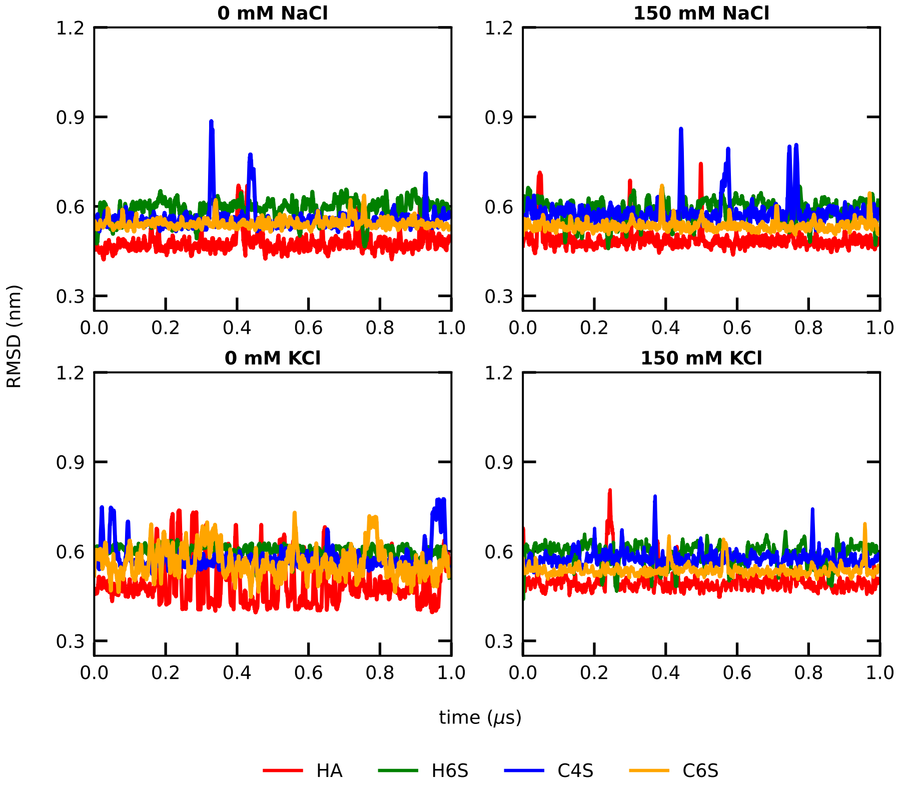

Figure S2. Evolution of the root mean square deviation (RMSD) of the GAGs at 0 mM (**left**) and 150 mM (**right**) salt concentrations (see label above each panel). The color key for the GAGs is given at the bottom of the figure.

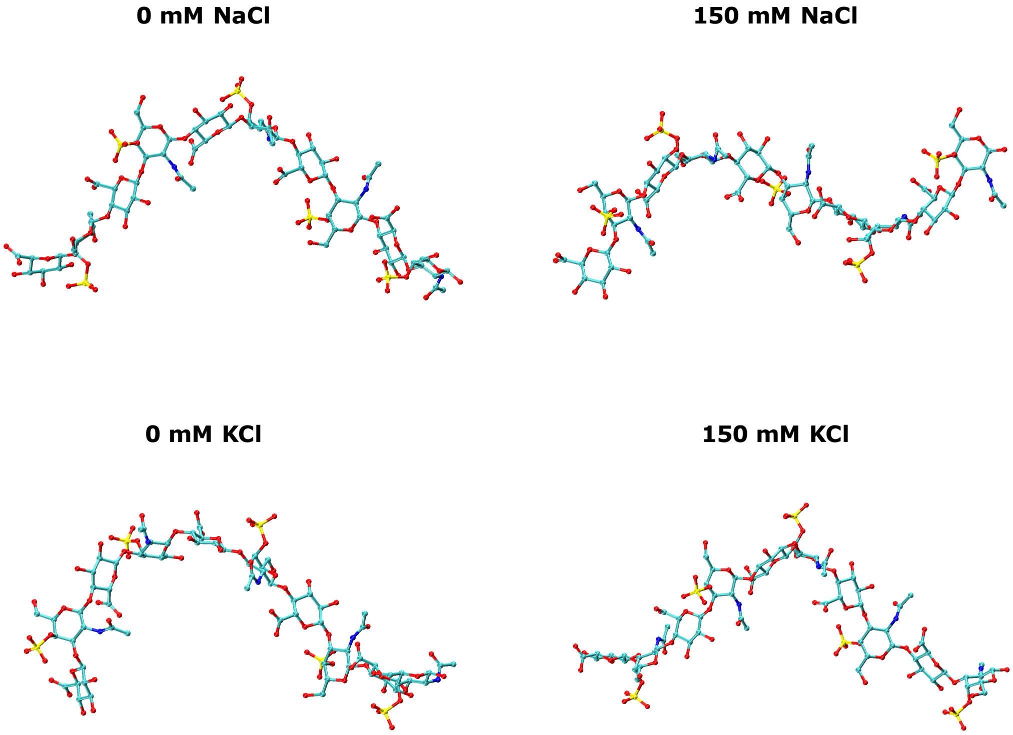

Figure S3. Snapshots illustrating the structures of C4S with RMSD > 0.7 nm at 0 mM (**left**) and 150 mM (**right**) salt concentrations.

Table S1: The average values of R_{ee}, N_{HB}, ϕ and ψ pairs for $Linkage_1$ and $Linkage_2$ of the C4S structures with RMSD $>$ 0.7 nm (mean $\pm$ standard error). The corresponding values of the initial C4S structures used in the MD simulations are provided too.

System	Structure	R_{ee}(nm)	N_{HB}	$Linkage_1$(°)	$Linkage_2$(°)
0 mM NaCl	starting	4.4	108.0	(−131.6, −150.8)	(−104.5, 78.7)
		3.1 ± 0.0	107.7 ± 0.2	(−57.0 ± 0.3, −118.7 ± 0.1)	(−78.4 ± 0.2, 125.6 ± 0.4)
150 mM NaCl	starting	4.4	108.0	(−139.4, −146.9)	(−102.8, 91.1)
		3.1 ± 0.0	107.7 ± 0.1	(−62.6 ± 0.2, 118.6 ± 0.1)	(−79.1 ± 0.1, 115.8 ± 0.5)
0 mM KCl	starting	4.3	121.0	(−138.3, −143.6)	(−105.8, 83.3)
		3.0 ± 0.0	110.0 ± 0.1	(-62.7 ± 0.2, −117.3 ± 0.1)	(−77.1 ± 0.1, 106.4 ± 0.6)
150 mM KCl	starting	4.4	115.0	(−124.6, −145.6)	(−104.4, 77.7)
		3.2 ± 0.0	105.9 ± 0.1	(−61.1 ± 0.3, −118.5 ± 0.1)	(−78.8 ± 0.1, 119.6 ± 0.5)

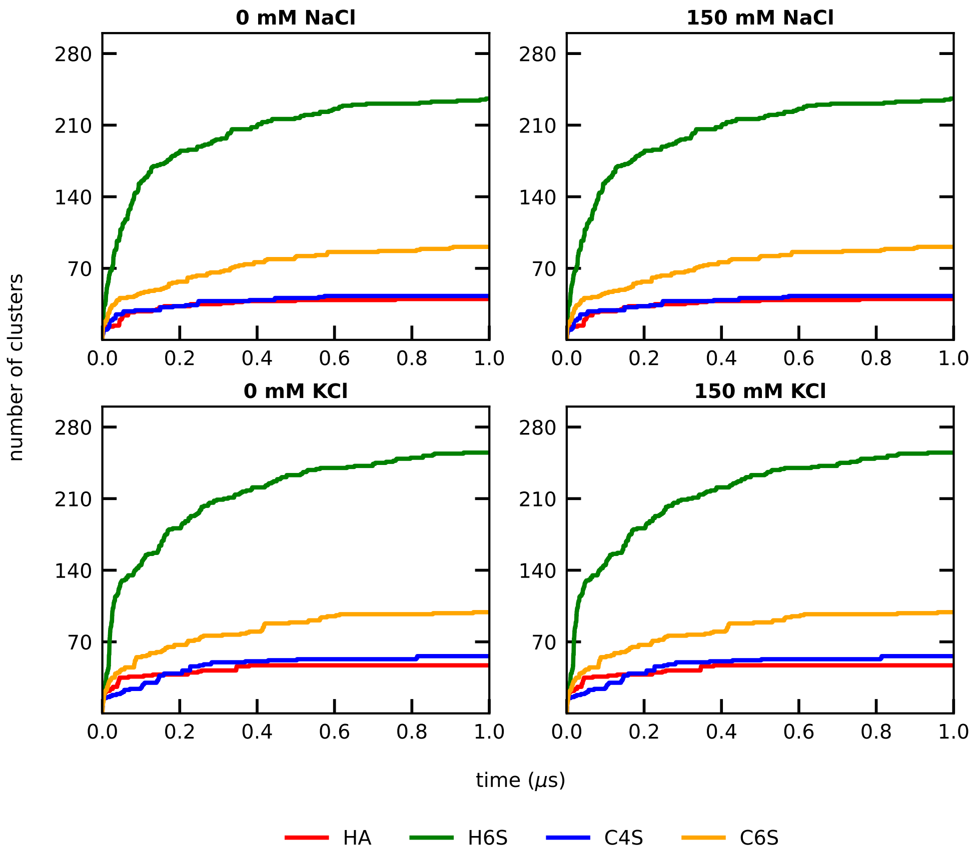

Figure S4. Evolution of the number of conformational clusters at $RMSD_{cutoff}$ = 0.3 nm for the different GAGs (see color key at the bottom) at 0 mM (**left**) and 150 mM (**right**) salt concentrations (see label above each panel).

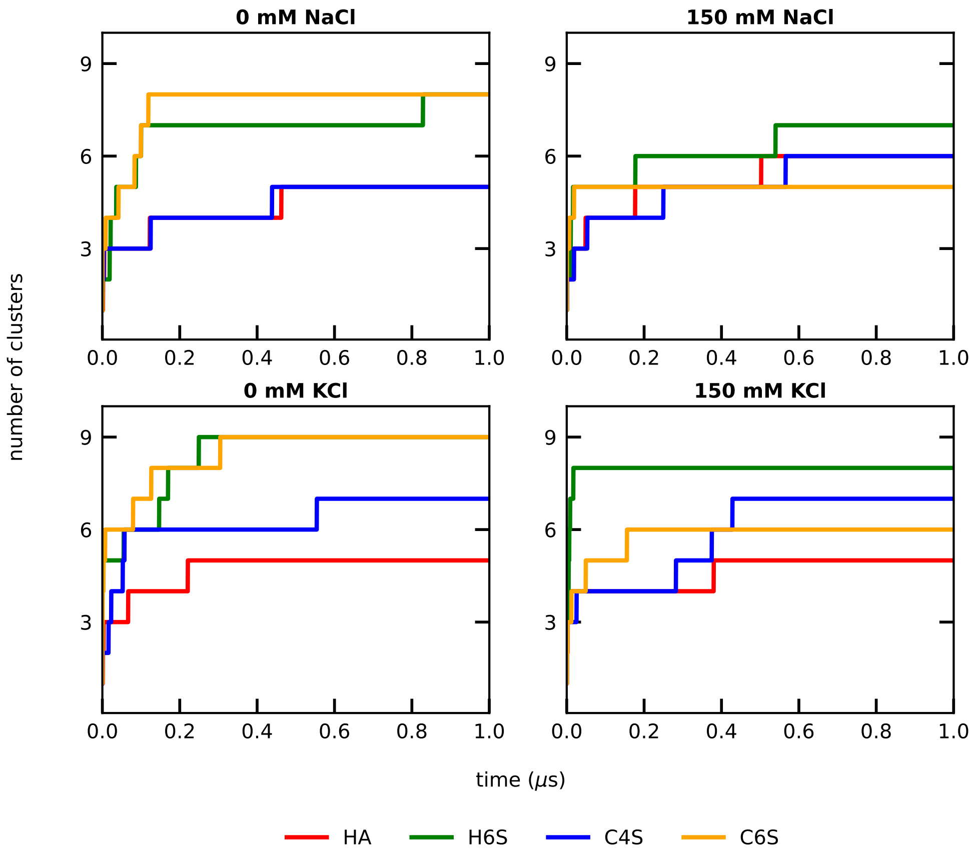

Figure S5. Evolution of the number of conformational clusters at $RMSD_{cutoff}$ = 0.5 nm for the different GAGs (see color key at the bottom) at 0 mM (**left**) and 150 mM (**right**) salt concentrations (see label above each panel).

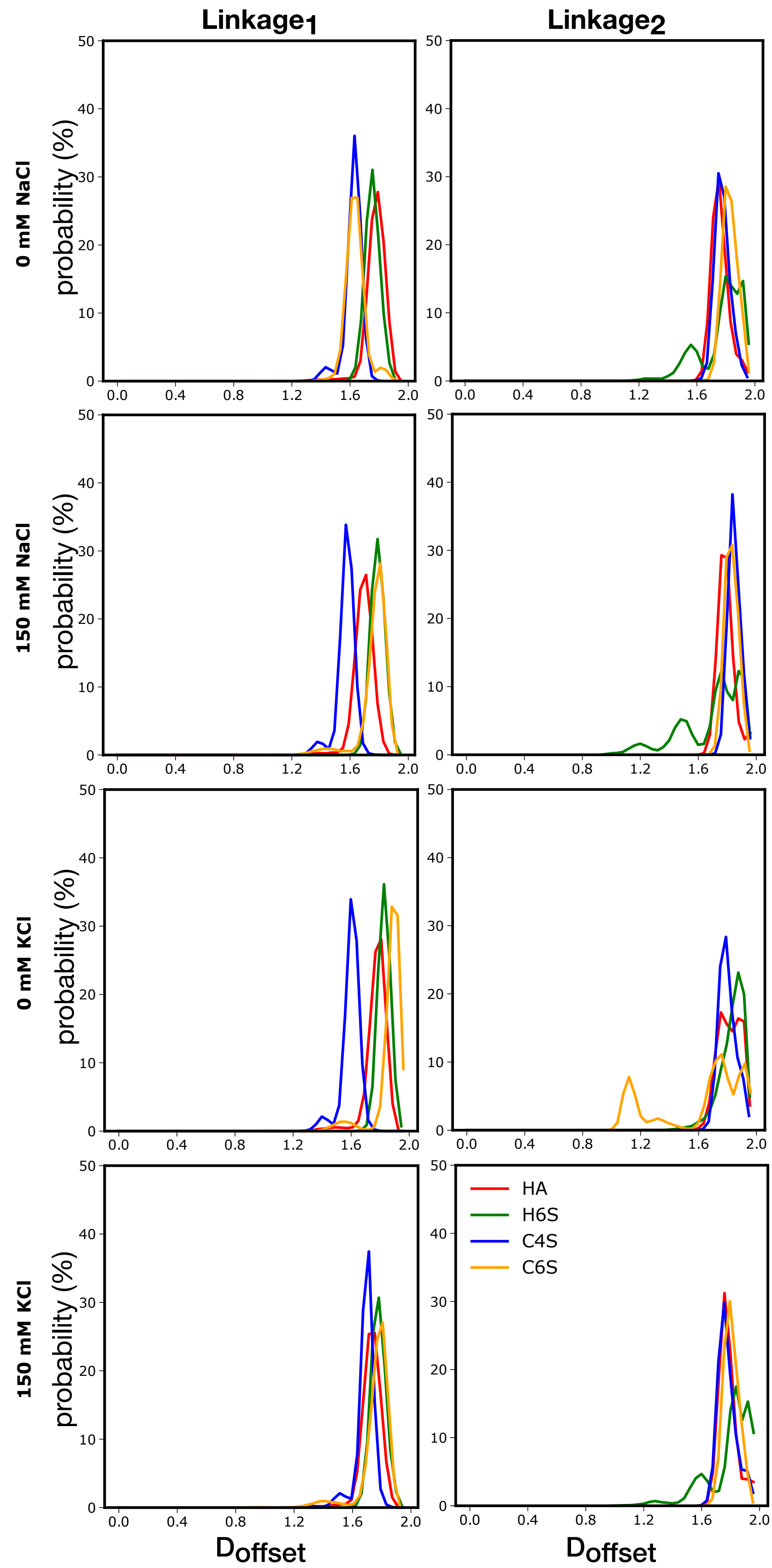

Figure S6. Distribution of the dihedral angle offset function, D_{offset}, for $Linkage_1$ (**left**) and $Linkage_2$ (**right**). The different GAGs are shown with different colors (see key in the lowest right panel). The salt concentrations are shown from top to bottom as 0 mM NaCl, 150 mM NaCl, 0 mM KCl, and 150 mM KCl.

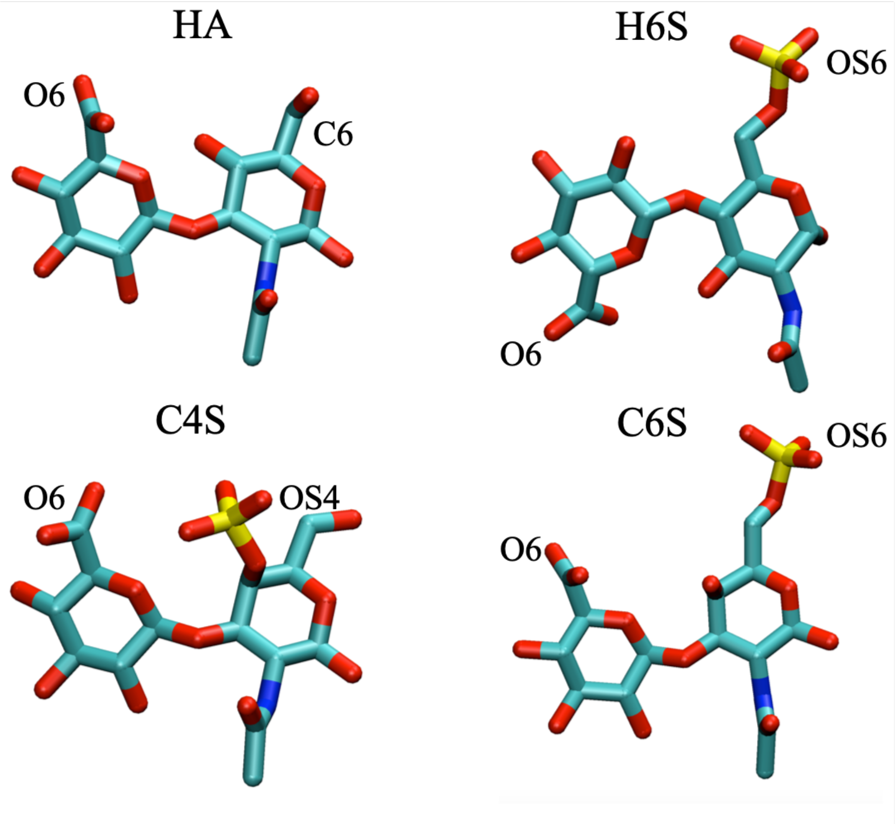

Figure S7. Identification of atoms relevant for the RDF calculations.

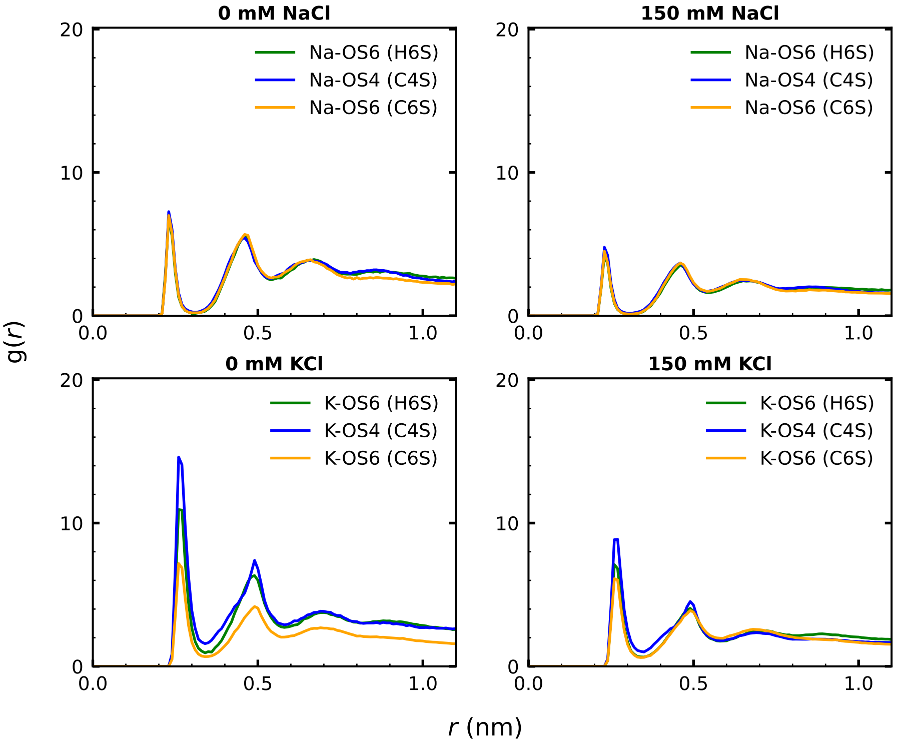

Figure S8. RDF curves representing the interactions between OSO_3^- and the cations Na^+ (top) and K^+ (**bottom**) at 0 mM (**left**) and 150 mM (**right**) salt concentration (see label above each panel). The RDF curves are averaged over the three oxygen atoms bound to the sulphur atom (see Figure S7 for the atom naming) and over the five OSO_3^- per GAG.

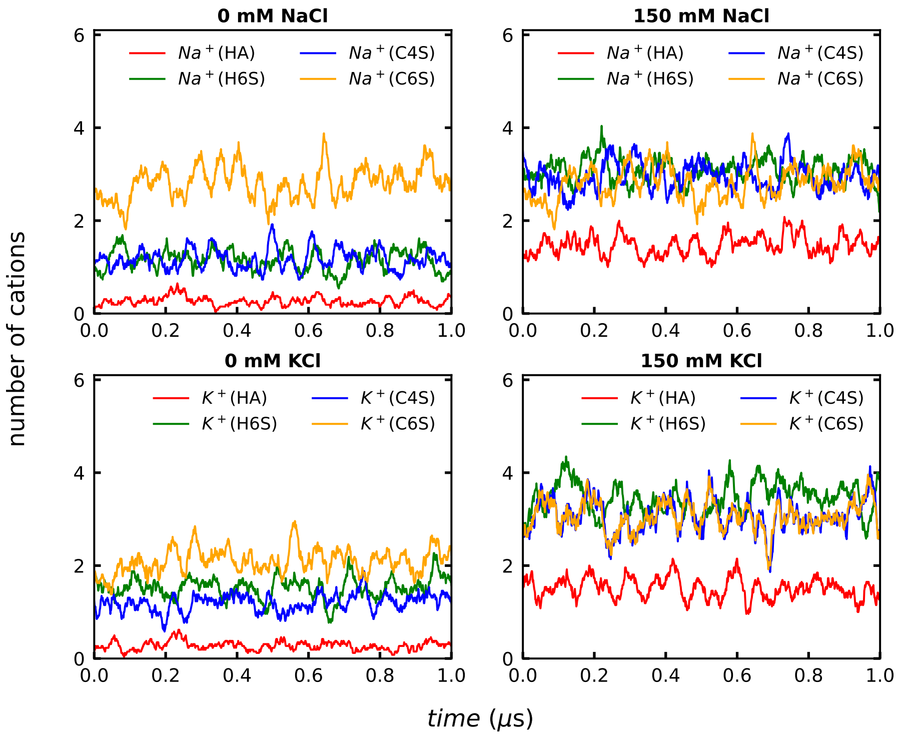

Figure S9. Evolution of the number of Na^+ (top) and K^+ (bottom) ions within 0.5 nm of the GAGs at 0 mM (**left**) and 150 mM (**right**) salt concentration (see label above each panel).

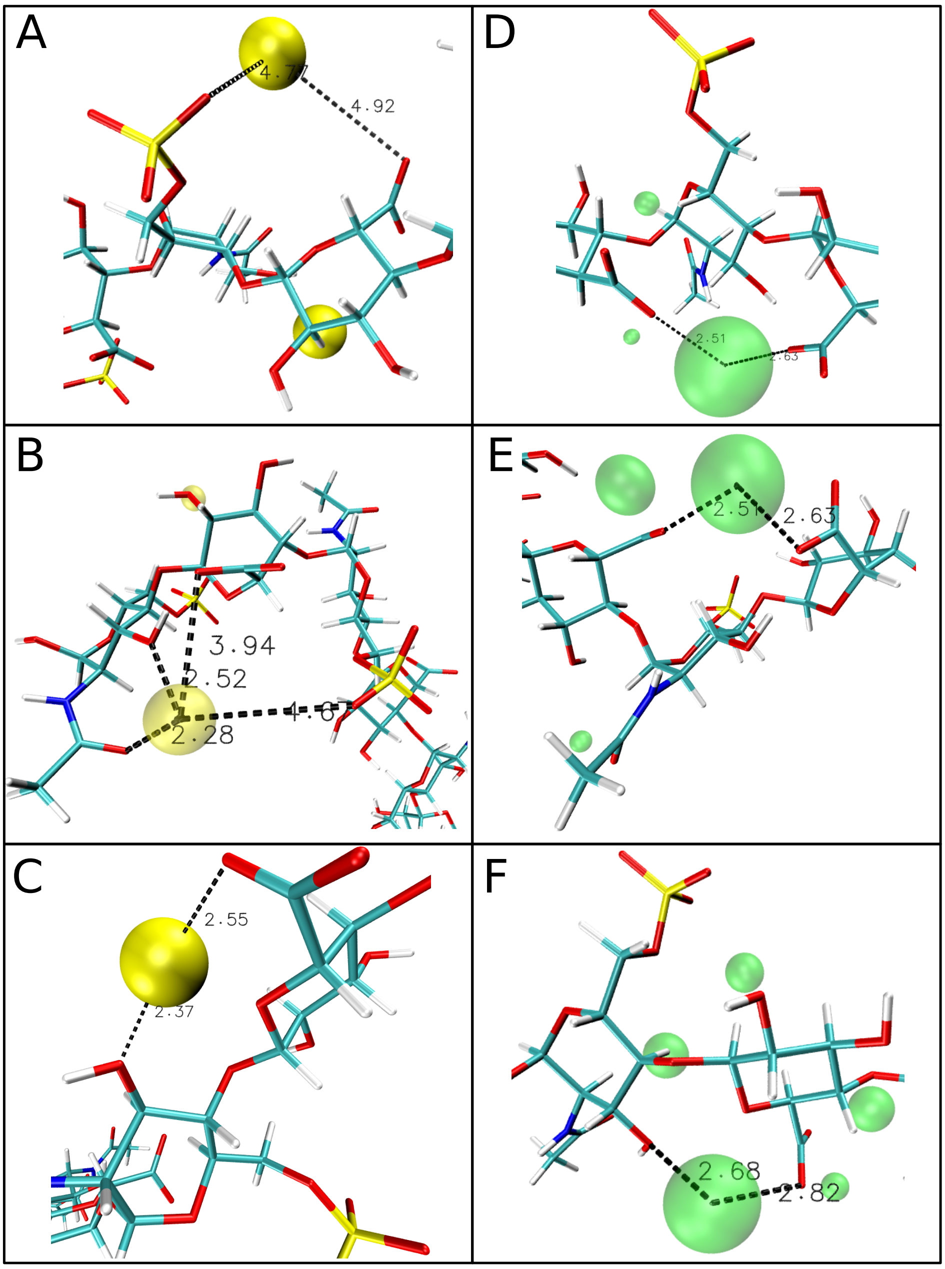

Figure S10. Snapshots illustrating the GAG interactions of H6S with Na^+ (**left**) and K^+ (**right**). The sodium ion is often involved in solvent separated COO^-–K^+–OSO_3^- bridging (**A**,**B**), whereas the potassium ion prefers direct binding via COO^-–K^+–COO^- bridging (**D**,**E**). Both cations are equally involved in direct binding via COO^-–cation–OH bridging (**C**,**F**). (Distances shown are in Å).

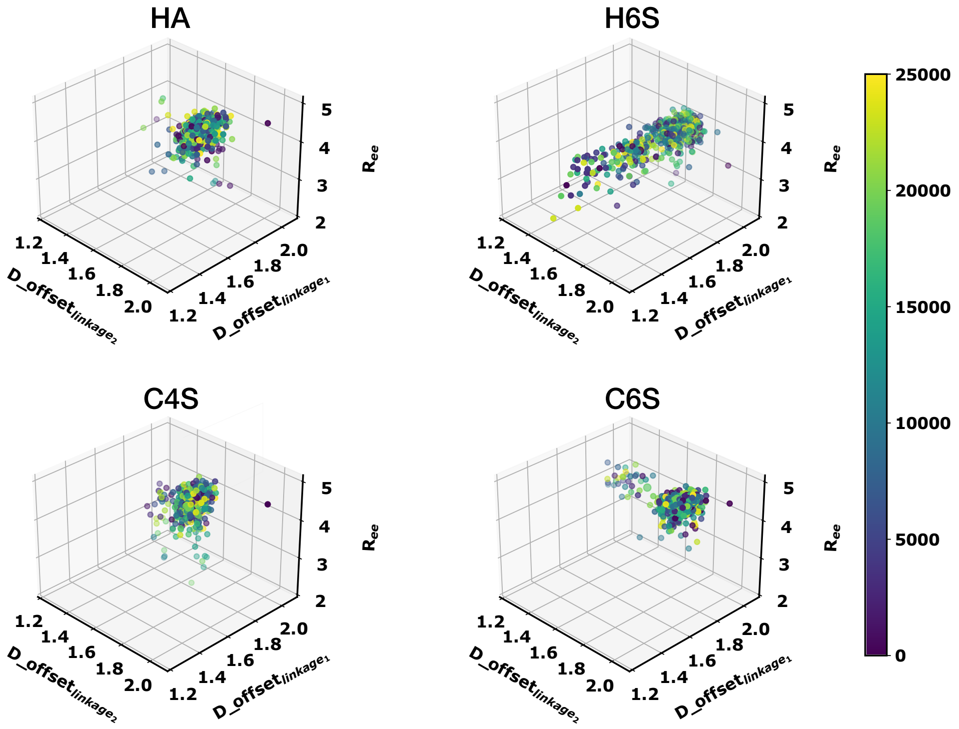

Figure S11. Distribution of the R_{ee} and D_{offset} values for $Linkage_1$ and $Linkage_2$ obtained for the GAG systems at 150 mM NaCl. Results for 25,000 MD snapshots are shown and the coloring of the dots reflects the simulation time, with dark blue for $t = 0$ (first snapshot) and yellow for $t = 1$ μs (last snapshot).

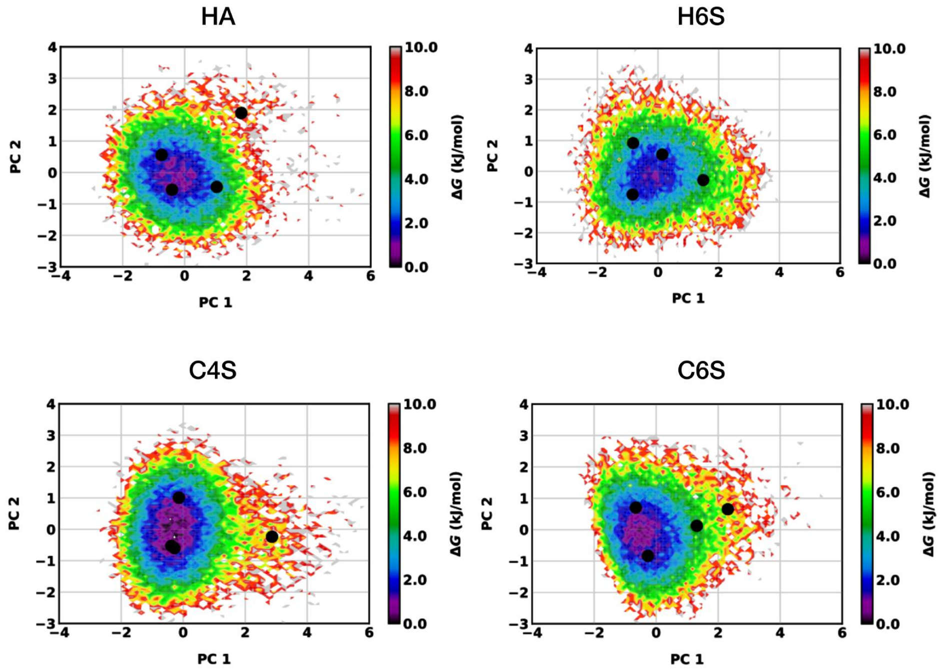

Figure S12. The free energy landscapes projected onto PC1 and PC2 for the GAG systems simulated in the presence of 150 mM NaCl.

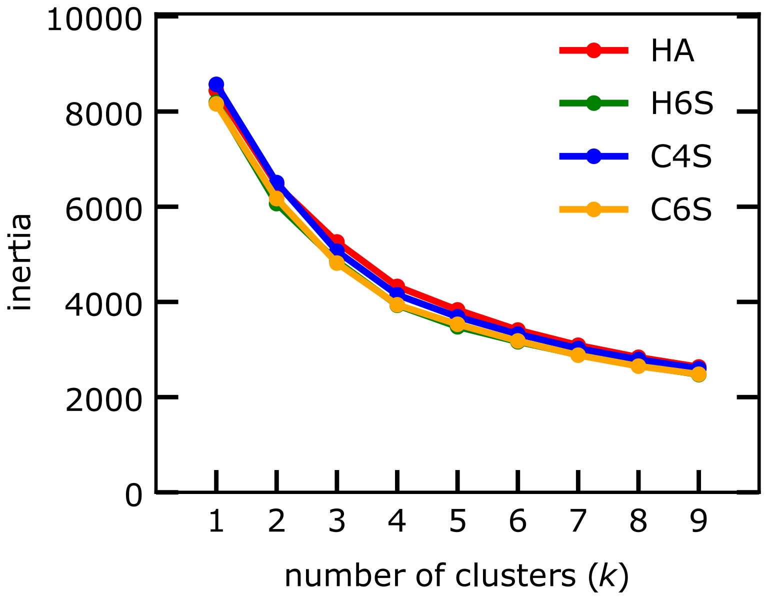

Figure S13. Determination of the optimal number of clusters, *k*, by plotting the inertia for increasing *k*. Results are shown for the GAGs simulated in the presence of 150 mM NaCl.

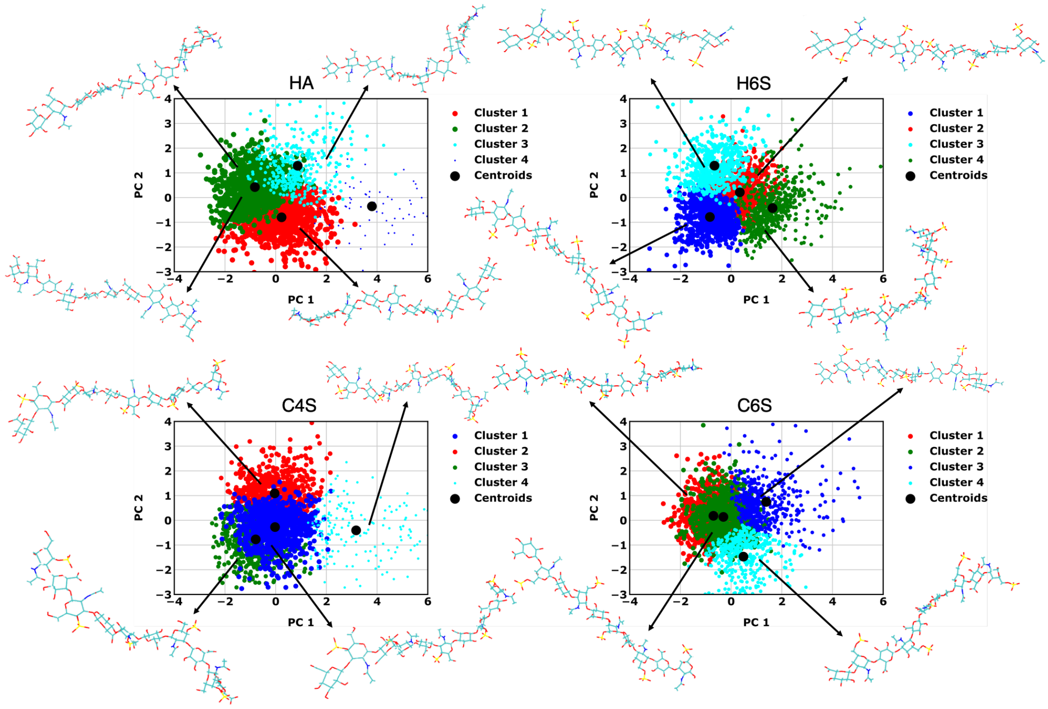

Figure S14. Projection of the MD trajectories obtained at 0 mM NaCl onto the first two principal components for the different GAGs (see label above each panel). The conformations are segregated into four clusters, which are represented by different colours. Structures corresponding to the centroid structures are shown.

Table S2: The values of R_{ee}, N_{HB}, ϕ and ψ pairs for $Linkage_1$ and $Linkage_2$ of the centroid structures of the four clusters (populations are provided below) obtained for the GAGs at 0 mM NaCl. The corresponding values of the initial structures used in the MD simulations are provided too. (mean ± standard error)

System	Structure	%	R_{ee}(nm)	N_{HB}	$Linkage_1$(°)	$Linkage_2$(°)
HA	starting		4.5	91.0	(−117.5, −150.5)	(−104.4, 76.3)
	cluster 1	41.8	4.0 ± 0.0	84.6 ± 0.1	(−71.1 ± 0.2, −124.8 ± 0.2)	(−77.2 ± 0.2, 128.1 ± 0.2)
	cluster 2	40.9	4.2 ± 0.0	77.3 ± 0.1	(−74.2 ± 0.2, −129.5 ± 0.2)	(−78.1 ± 0.2, 124.3 ± 0.3)
	cluster 3	14.4	3.8 ± 0.0	82.2 ± 0.2	(−71.0 ± 0.3, −124.6 ± 0.4)	(−80.3 ± 0.3, 99.9 ± 1.0)
	cluster 4	2.8	3.6 ± 0.1	82.6 ± 0.6	(−63.1 ± 1.5, −90.0 ± 2.9)	(−76.9 ± 0.8, 122.4 ± 1.9)
H6S	starting		4.4	109.0	(−118.1, 87.5)	(49.4, 59.8)
	cluster 1	31.0	4.2 ± 0.0	110.7 ± 0.2	(−71.5 ± 0.2, 123.1 ± 0.2)	(79.3 ± 0.3, 48.1 ± 1.0)
	cluster 2	27.7	4.3 ± 0.0	104.1 ± 0.2	(−76.9 ± 0.2, 116.9 ± 0.3)	(81.6 ± 0.3, 41.6 ± 1.0)
	cluster 3	21.5	3.6 ± 0.0	104.8 ± 0.2	(−74.1 ± 0.2, 121.1 ± 0.3)	(80.8 ± 0.4, 50.6 ± 1.4)
	cluster 4	19.8	4.3 ± 0.0	103.7 ± 0.2	(−74.1 ± 0.2, 117.9 ± 0.4)	(85.7 ± 0.2, −12.6 ± 0.8)
C4S	starting		4.4	108.0	(−131.6, −150.8)	(−104.5, 78.7)
	cluster 1	35.8	4.0 ± 0.0	103.6 ± 0.1	(−67.2 ± 0.2, −121.8 ± 0.2)	(−77.5 ± 0.2, 127.6 ± 0.2)
	cluster 2	31.1	4.0 ± 0.0	113.2 ± 0.1	(−67.7 ± 0.2, −121.5 ± 0.2)	(−76.6 ± 0.2, 126.8 ± 0.3)
	cluster 3	26.0	4.1 ± 0.0	107.1 ± 0.2	(-67.9 ± 0.2, −122.4 ± 0.2)	(−80.2 ± 0.2, 113.3 ± 0.3)
	cluster 4	7.1	3.3 ± 0.0	108.0 ± 0.4	(−49.4 ± 0.7, −119.4 ± 0.4)	(−78.8 ± 0.4, 125.1 ± 0.8)
C6S	starting		4.5	109.0	(−121.5, −89.3)	(−100.5, 80.9)
	cluster 1	34.2	4.4 ± 0.0	112.3 ± 0.1	(−73.5 ± 0.2, −142.0 ± 0.3)	(−72.6 ± 0.2, 119.4 ± 0.3)
	cluster 2	31.4	4.4 ± 0.0	102.8 ± 0.1	(−73.7 ± 0.2, −140.9 ± 0.4)	(−74.4 ± 0.2, 117.1 ± 0.3)
	cluster 3	18.0	4.1 ± 0.0	108.8 ± 0.2	(−72.0 ± 0.4, −136.7 ± 0.7)	(−76.8 ± 0.3, 97.8 ± 0.8)
	cluster 4	16.4	4.2 ± 0.0	107.3 ± 0.2	(−75.7 ± 0.3, −113.8 ± 1.3)	(−74.2 ± 0.3, 119.9 ± 0.5)

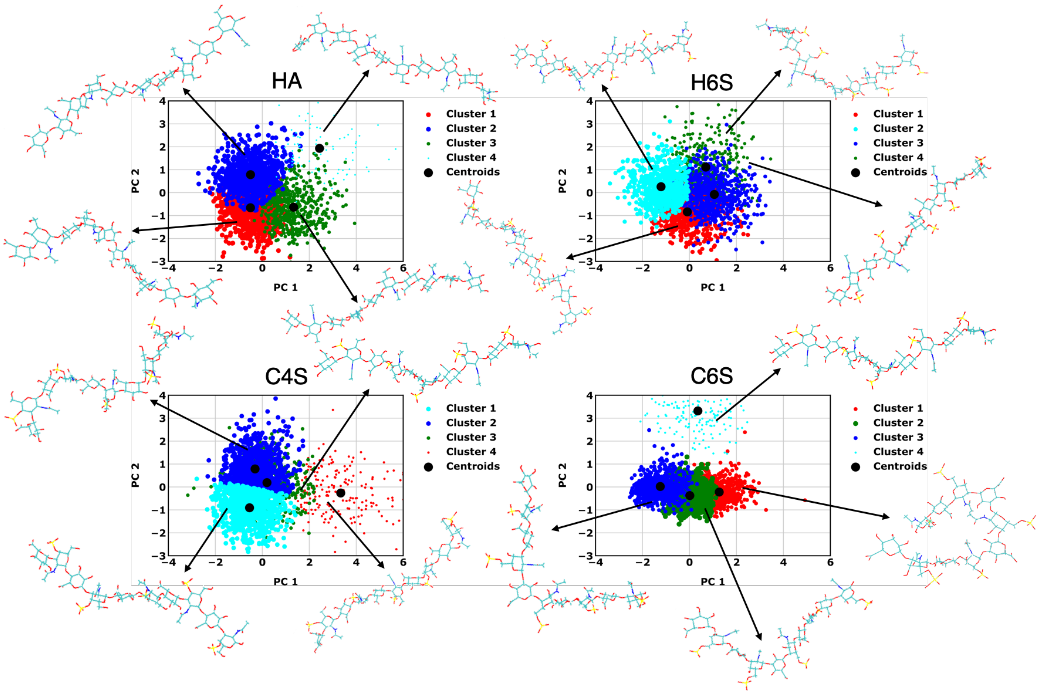

Figure S15. Projection of the MD trajectories obtained at 0 mM KCl onto the first two principal components for the different GAGs (see label above each panel). The conformations are segregated into four clusters, which are represented by different colours. Structures corresponding to the centroid structures are shown.

Table S3: The values of R_{ee}, N_{HB}, ϕ and ψ pairs for $Linkage_1$ and $Linkage_2$ of the centroid structures of the four clusters (populations are provided below) obtained for the GAGs at 0 mM KCl. The corresponding values of the initial structures used in the MD simulations are provided too. (mean ± standard error)

System	Structure	%	R_{ee}(nm)	N_{HB}	$Linkage_1$(°)	$Linkage_2$(°)
	starting		4.5	88.0	(−121.9, −149.3)	(−104.3, 78.9)
	cluster 1	38.6	4.2 ± 0.0	80.1 ± 0.1	(−76.6 ± 0.2, −128.4 ± 0.2)	(−75.3 ± 0.2, 104.3 ± 0.7)
HA	cluster 2	37.6	4.2 ± 0.0	80.1 ± 0.1	(−76.6 ± 0.2, −128.4 ± 0.2)	(−75.3 ± 0.2, 104.3 ± 0.7)
	cluster 3	20.3	3.3 ± 0.0	84.4 ± 0.2	(−75.3 ± 0.3, −125.5 ± 0.3)	(−75.5 ± 0.3, 72.9 ± 0.9)
	cluster 4	3.5	3.7 ± 0.1	84.4 ± 0.6	(−69.6 ± 1.1, −85.8 ± 1.9)	(−72.9 ± 0.8, 102.0 ± 3.2)
	starting		4.3	94.0	(−105.4, 85.9)	(43.0, 60.9)
	cluster 1	30.0	4.2 ± 0.0	106.2 ± 0.2	(−77.2 ± 0.2, 115.0 ± 0.2)	(73.8 ± 0.3, 67.2 ± 0.7)
H6S	cluster 2	29.6	4.1 ± 0.0	112.5 ± 0.2	(−71.8 ± 0.2, 122.1 ± 0.2)	(73.4 ± 0.3, 73.9 ± 0.7)
	cluster 3	24.2	3.5 ± 0.0	104.8 ± 0.2	(−74.9 ± 0.2, 117.8 ± 0.3)	(75.1 ± 0.3, 79.8 ± 0.9)
	cluster 4	16.2	4.0 ± 0.0	106.3 ± 0.2	(−74.3 ± 0.3, 118.0 ± 0.3)	(86.5 ± 0.4, 74.3 ± 2.2)
	starting		4.3	121.0	(−138.3, −143.6)	(−105.8, 83.3)
	cluster 1	36.1	4.0 ± 0.0	113.7 ± 0.1	(−69.0 ± 0.2, −120.0 ± 0.2)	(−75.0 ± 0.2, 128.0 ± 0.2)
C4S	cluster 2	35.8	4.0 ± 0.0	104.5 ± 0.1	(−68.3 ± 0.2, −120.3 ± 0.2)	(−75.8 ± 0.2, 125.0 ± 0.2)
	cluster 3	20.5	4.0 ± 0.0	110.2 ± 0.2	(−69.7 ± 0.3, −120.4 ± 0.2)	(−78.4 ± 0.2, 116.2 ± 0.7)
	cluster 4	7.6	3.0 ± 0.0	108.7 ± 0.4	(−52.3 ± 0.8, −117.0 ± 0.5)	(−76.3 ± 0.4, 95.0 ± 1.6)
	starting		4.6	120.0	(−81.2, −103.0)	(162.8, 45.2)
	cluster 1	43.1	3.9 ± 0.0	108.7 ± 0.2	(−50.7 ± 0.3, −92.1 ± 0.2)	(−14.0 ± 1.2, 59.5 ± 0.4)
C6S	cluster 2	26.1	3.7 ± 0.0	108.4 ± 0.2	(−50.0 ± 0.3, −91.8 ± 0.3)	(−109.2 ± 0.6, 58.0 ± 0.6)
	cluster 3	24.7	3.0 ± 0.0	108.1 ± 0.2	(−49.3 ± 0.3, −91.0 ± 0.3)	(−18.2 ± 1.5, 57.7 ± 0.6)
	cluster 4	6.1	3.6 ± 0.0	108.0 ± 0.4	(−51.6 ± 0.7, −25.0 ± 1.1)	(−41.2 ± 4.2, 56.4 ± 1.3)

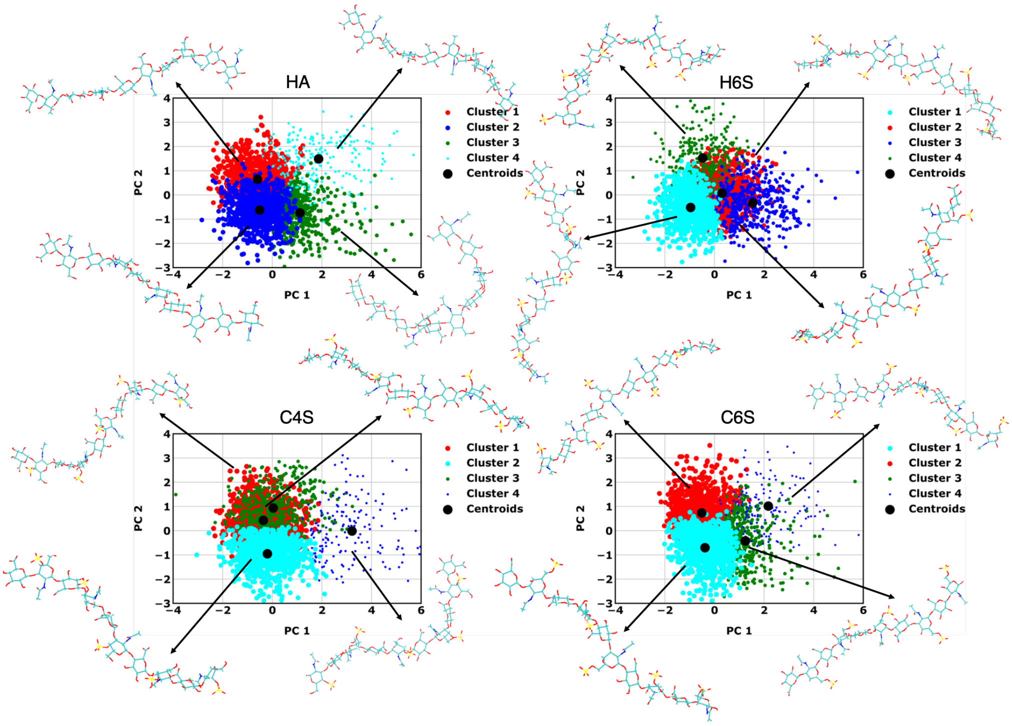

Figure S16. Projection of the MD trajectories obtained at 150 mM KCl onto the first two principal components for the different GAGs (see label above each panel). The conformations are segregated into four clusters, which are represented by different colours. Structures corresponding to the centroid structures are shown.

Table S4: The values of R_{ee}, N_{HB}, ϕ and ψ pairs for $Linkage_1$ and $Linkage_2$ of the centroid structures of the four clusters (populations are provided below) obtained for the GAGs at 150 mM KCl. The corresponding values of the initial structures used in the MD simulations are provided too. (mean ± standard error)

System	Structure	%	R_{ee}(nm)	N_{HB}	$Linkage_1$(°)	$Linkage_2$(°)
HA	starting		4.5	89.0	(−125.9, −148.0)	(−99.2, 76.3)
	cluster 1	35.6	4.2 ± 0.0	76.0 ± 0.1	(−73.1 ± 0.2, −128.1 ± 0.2)	(−79.2 ± 0.2, 124.0 ± 0.3)
	cluster 2	33.4	4.2 ± 0.0	84.1 ± 0.1	(−74.2 ± 0.2, −128.4 ± 0.2)	(−76.6 ± 0.2, 125.0 ± 0.2)
	cluster 3	22.5	3.8 ± 0.0	81.9 ± 0.2	(−66.0 ± 0.3, −117.0 ± 0.6)	(−78.4 ± 0.3, 128.0 ± 0.4)
	cluster 4	8.6	3.5 ± 0.0	81.7 ± 0.3	(−71.7 ± 0.5, −123.4 ± 0.5)	(−78.7 ± 0.5, 85.6 ± 0.9)
H6S	starting		4.4	106.0	(−112.5, 85.3)	(52.8, 57.5)
	cluster 1	34.4	4.3 ± 0.0	108.4 ± 0.2	(−71.7 ± 0.2, 123.6 ± 0.2)	(79.6 ± 0.3, 43.8 ± 0.9)
	cluster 2	34.2	4.3 ± 0.0	100.7 ± 0.2	(−76.0 ± 0.2, 117.6 ± 0.3)	(82.0 ± 0.3, 40.0 ± 0.8)
	cluster 3	17.8	4.3 ± 0.0	100.8 ± 0.3	(−73.9 ± 0.3, 118.4 ± 0.3)	(85.9 ± 0.4, −16.9 ± 1.0)
	cluster 4	13.6	3.8 ± 0.0	103.8 ± 0.3	(−74.8 ± 0.3, 117.6 ± 0.6)	(81.0 ± 0.5, 30.1 ± 1.6)
C4S	starting		4.4	115.0	(−124.6, −145.6)	(−104.4, 77.7)
	cluster 1	39.8	4.0 ± 0.0	101.1 ± 0.1	(−67.2 ± 0.2, −121.7 ± 0.2)	(−78.2 ± 0.2, 125.3 ± 0.2)
	cluster 2	36.0	3.9 ± 0.0	110.5 ± 0.1	(−67.0 ± 0.2, −121.1 ± 0.2)	(−77.3 ± 0.2, 128.0 ± 0.2)
	cluster 3	17.6	3.9 ± 0.0	107.1 ± 0.2	(−68.0 ± 0.3, −121.9 ± 0.3)	(−80.2 ± 0.3, 100.4 ± 0.8)
	cluster 4	6.6	3.3 ± 0.0	105.2 ± 0.4	(−44.0 ± 0.8, −119.2 ± 0.4)	(−78.2 ± 0.5, 115.6 ± 1.6)
C6S	starting		4.5	106.0	(−121.8, −157.6)	(−104.8, 80.4)
	cluster 1	38.7	4.4 ± 0.0	101.6 ± 0.1	(−74.4 ± 0.2, −140.9 ± 0.3)	(−75.2 ± 0.2, 116.2 ± 0.3)
	cluster 2	37.8	4.4 ± 0.0	110.2 ± 0.1	(−74.2 ± 0.2, −140.5 ± 0.3)	(−72.7 ± 0.2, 119.6 ± 0.3)
	cluster 3	17.9	3.9 ± 0.0	105.1 ± 0.2	(−72.0 ± 0.3, −136.0 ± 0.4)	(−76.4 ± 0.3, 108.5 ± 0.9)
	cluster 4	5.6	4.3 ± 0.0	106.0 ± 0.5	(−75.0 ± 0.7, −78.8 ± 1.4)	(−74.4 ± 0.5, 118.0 ± 1.0)
